# First report of AChE1 (G119S) mutation and multiple resistance mechanisms in *Anopheles gambiae* s.s. in Nigeria

**DOI:** 10.1038/s41598-020-64412-7

**Published:** 2020-05-04

**Authors:** Ifeoluwa Kayode Fagbohun, Emmanuel Taiwo Idowu, Olubunmi Adetoro Otubanjo, Taiwo Samson Awolola

**Affiliations:** 1 Department of Zoology, University of Lagos, Akoka, Yaba, Lagos Nigeria; 2 Vector research unit, Nigeria Institute Medical Research, Yaba, Lagos Nigeria

**Keywords:** Entomology, Mutation

## Abstract

Susceptibility and PBO synergist bioassays were done using 3–5 days old female *Anopheles* mosquito collected from Lagos State, Nigeria with WHO test papers DDT (4%), permethrin (0.75%), Bendiocarb (1%) and PBO (4%) according to standard procedures. The activities of cytochrome P450s, glutathione S-transferase and carboxylesterases were determined using biochemical assays. The presence of *kdr-w*, *kdr-e* and *Ace-1*^*R*^ mutations were examined using molecular assays. Resistance to DDT and permethrin in *An gambiae* s.s from the four Local Government Areas (LGAs) was recorded while suspected resistance to bendiocarb was recorded in mosquitoes from Alimosho and Kosofe LGAs. PBO synergist reduced the knockdown time and also recorded significantly (P < 0.05) higher 24 hrs percentage mortality compared to non-synergized bioassays. Increased activities of detoxifying enzymes was recorded in wild mosquito compared to the insecticides susceptible laboratory strain and this was significant (P < 0.05) in P450s, esterase α and β. *Kdr-w* was detected in *An. gambiae* s.s from all the LGAs, *kdr-e* (L1014S) was detected in Alimosho, Kosofe and Ibeju-Lekki, while the *Ace-1*^*R*^ gene was detected in Alimosho and Kosofe. Results from this study provide evidence for resistance of *An. gambiae* from Lagos State to multiple classes of neurotoxic insecticides with multiple resistance mechanisms to these insecticides.

## Introduction

Malaria remains a major public health problem in sub-Saharan Africa, the World Health Organization (WHO) estimated 219 million cases and 430,000 mortality attributed to malaria globally in 2017 with Nigeria carrying the highest (19%) burden of the estimated death cases^1^. The global fight against malaria highlighted in the United Nations sustainable development goals (SDGs) goal 3.3 aligns with the WHO global technical strategy for malaria, the roll back malaria partnership (RBM) action and investment to defeat malaria (AIM). Specifically, the goal is to reduce the global malaria mortality and incidence rates by 90% in the year 2030 using 2015 as the baseline^2–4^.

The use of insecticide based vector control measures is vital for the reduction of malaria incidence globally. Scaling up the use of long lasting insecticides treated nets (LLINs) and indoor residual spraying (IRS) has been significantly crucial in the protection of several individuals in endemic areas especially infants and pregnant women^1,5,6^.

The effectiveness and efficacy of the few insecticides approved for the control of malaria vectors by WHO has been greatly hampered by the advent of insecticide resistance. In Nigeria, Pyrethriods and DDT resistance has been reported in several parts of the country^7–12^. Insecticides resistance in malaria vectors can result from one or combination of behavioral changes, morphological modifications, target site mutation and metabolism by detoxifying enzymes. Knockdown resistance (L1014F and L1014S) has first been described in Pyrethriods resistance malaria vector^13,14^. Subsequently, these mutations have been identified in Pyrethriods and DDT resistance *Anopheles* in different parts of Africa^10,15–19^. The increase in the detoxifying and/or sequestering activities of cytochrome P450s, GSTs and Esterase have been associated with mosquitoes resistance to insecticides^20–22^. Piperonyl butoxide is a non-toxic synergist but has been proven to suppress DDT and Pyrethriods resistance in malaria vectors under laboratory conditions^23–25^. Likewise, in several endemic areas in sub-Saharan Africa, PBO incorporated into LLINs has also shown to be more efficacious and effective in controlling malaria vectors, thereby, reducing malaria incidence^26–29^.

The need for regular monitoring susceptibility status and resistance mechanisms of malaria vectors in endemic areas becomes more important given the changing susceptibility status of *Anopheles* mosquitoes. Therefore, this study provides information on, the efficacy of PBO synergist based insecticides control measures in the management of DDT and permethrin resistant malaria vectors, and the presence of molecular resistance mechanism (L1014F, L1014S and G119S) in Lagos State, Nigeria.

## Results

### Insecticide susceptibility status of *An. gambiae* s.l. in lagos state, nigeria

Resistance to DDT and permethrin was recorded in *An. gambiae* s.l. in all the LGAs with 24hrs percentage mortality ranging from 9 to 55 and 22 to 70 for DDT and permethrin respectively. The estimated knockdown time for 50% (KDT_50_) of the mosquito assayed with DDT varied from 55 minutes in Badagry to 996.9 minutes in Alimosho LGA. While the estimated knockdown time for 95% (KDT_95_) of the mosquitoes from Lagos State exposed to permethrin was 3864.9 minutes for Alimosho LGA, 9361 minutes for Ibeju-Lekki LGA, 136.6 minutes for Badagry and 3127.1 minutes for Kosofe LGA (Table 1). Suspected resistance to bendiocarb was record in mosquitoes form Kosofe and Alimosho LGAs with KDT_50_ of 33.1 and 23.3 minutes respectively, while full susceptibility was observed in Ibeju-Lekki and Badagry LGAs (Table 2).

**Table 1:** KDT_50_, KDT_95_ values and percentage mortality of *An. gambiae* s.l. from LGAs of Lagos State exposed to DDT, permethrin and PBO synergist.

Location		Number exposed	KDT_50_ (95% cl)	KDT_95_ (95% cl)	Mortality (%)	Resistance status	P value
Alimosho	DDT	100	996.9(261.1–413030.8)	28091 (2024.1–4.4 × 10^9^)	9	R	0.000
	PBO + DDT	100	47(42.6–53)	204.6(142–273.4)	79		
	PER	100	180.5 (108.5–533.1)	3864.9 (1026.7–79822.7)	42	R	0.000
	PBO + PER	100	37.2(34.2–40.8)	136.5(110.5–181.3)	88		
Ibeju-Lekki	DDT	100	226.3(131.4–785.2)	1901.8(607.1–27507.7)	18	R	0.000
	PBO + DDT	100	38.4(35.3–42,2)	140.4(113.2–187.7)	88		
	PER	100	464(132–7.4 × 10^9^)	9361.7(679.6–2.7 × 10^19^)	22	R	0.000
	PBO + PER	100	25.5(23.6–27.4)	79.6(69–95.7)	96		
Badagry	DDT	100	55 (49.6–62.8)	193.8(148–284.5)	55	R	0.004
	PBO + DDT	100	48.1(44.4–52.8)	137(112.6–179.3)	90		
	PER	100	45.6 (42.2–50.1)	136.6 (112.3–177.8)	70	R	0.031
	PBO + PER	100	37.7(32.8–44)	83.7(65.7–140)	98		
Kosofe	PER	100	382.4(164.1–8117.5)	3127.1(631.3–1108705)	17	R	0.000
	PBO + PER	100	68.2(52.8–109.5)	381.4(194.2–1578.2)	87		
Kisumu	DDT	50	48.5(42.1–58.6)	195.2(145.2–353.2)	97		
	Permethrin	50	12(9.6–13.9)	20(17.4–31.4)	100		

Note: Mortality of >98% indicates Susceptibility, 97–90 Suspected resistance and <90% resistant^60^; S: susceptible, SR: suspected resistance, R: resistant, KDT: log probit estimates knockdown time, Kisumu: Laboratory susceptible strain, bioassay consist of 25 female *Anopheles* mosquitoes in four replicates. P is significant at P < 0.05.

**Table 2:** KDT_50_, KDT_95_ values and percentage mortality of *An. gambiae* s.l. from LGAs of Lagos State exposed to bendiocarb (0.1%).

	Kosofe	Alimosho	Ibeju-Lekki	Badagry	Kisumu
No. exposed	100	100	100	100	50
Knockdown at 10 min (%)	0	2	2	2	14
Knockdown at 30 min (%)	42	66	78	72	88
Knockdown at 60 min (%)	95	93	99	99	100
KDT_50_ (Min)	33.1(29.6–36.8)	23.3(19–27.7)	19.3(17.1–21.4)	20.5(18.1–22.9)	18.5(16.8–20.1)
KDT_95_ (Min)	60(52.3–77.8)	64.7(49.5–104.4)	45(38.6–55.7)	44.7(38–56.7)	39.2(34.6–46.5)
24 hr % mortality	96	96	100	100	100
Resistance Status	Suspected resistance	Suspected resistance	Susceptible	Susceptible	Susceptible

Note: KDT: log probit estimates knockdown time, Kisumu: Laboratory susceptible strain, bioassay consist of 25 female *Anopheles* mosquitoes in four replicates.

### Efficacy of PBO synergist on DDT and permethrin resistant *An. gambiae s.s*

Pre-exposure to PBO synergist significantly (P < 0.05) increases the 24hrs percentage mortality of *An. gambiae s.l*. in all the LGAs to DDT and permethrin. In addition, the estimated knockdown time for 50% and 95% of mosquitoes was also reduced when compared to that of the non-synergized bioassay, although only PBO + permethrin from Badagry was able to attain full susceptibility (Table 1). The percentage knockdown at various time intervals also shows that PBO synergized assay had faster knockdown rate in comparison to the non-synergized bioassays from the four LGAs (Fig. 2A–D).

### Activities of detoxifying enzymes in malaria vectors from lagos state, nigeria

The mean activities of detoxifying enzymes in field strains of *An. gambiae s.l*. in comparison with that of susceptible laboratory strain (Kisumu) is displayed in Fig. 3A–D. All enzymes including P450s, GSTs, esterase α and esterase β show higher level of activities in field strains compared to Kisumu strain. The difference in the enzymes activities of both the field and laboratory strains shows a statistical significance (P < 0.05) in the mean values for P450s, esterase α and esterase β especially from Kosofe and Alimosho LGAs.

### Detection of point mutation associated neurotoxic insecticides resistance in *An. gambiae* s.s. population from lagos state

Table 3 shows the allele frequency at the *kdr* (L1014S and L104F) and *Ace-1*^*R*^ (G119S) loci of *An. gambiae* in Lagos State. Homozygotes and heterozygotes resistance *kdr* west (L1014F) genotype were detected in all the sampled LGAs, with allele frequency ranging from 0.37 to 0.5. The distribution of L1014F mutation within the surveyed location did not significantly depart from the Hardy-Weinberg equilibrium (P > 0.05). *Kdr* east (L1014S) was also detected in Alimosho, Kosofe and Ibeju-Lekki LGAs with allele frequencies of 37%, 29% and 17% respectively and only heterozygotes resistance genotype identified. AChE1 mutation was detected in *An. gambiae* from Alimosho (32%) and Kosofe (36%) LGAs, meanwhile no *Ace-1*^*R*^ mutation was detected in Badagry and Ibeju-Lekki LGAs. The observed genotypic frequencies from *Ace-1*^*R*^ mutation in Kosofe and Alimosho LGAs were significantly different from Hardy-Weinberg expectations (P < 0.05).

**Table 3:** Frequency of *kdr* and Ache1 allele in *Anopheles gambiae* s.l. from Lagos State, Nigeria.

Mutation type	Location	Number (N)	Genotype (N)			Allele frequency	H-W P value
			RR	RS	SS		
*Kdr* west (L1014F)	Alimosho	72	18	7	0	0.5	0.32
	Kosofe	72	16	5	0	0.47	0.257
	Badagry	72	10	2	0	0.37	0.22
	Ibeju-Lekki	72	11	3	0	0.39	0.35
	**Total**	**288**	**55**	**19**	**0**		**0.16**
*Kdr* east (L1014S)	Alimosho	36	5	0	0	0.37	
	Kosofe	36	3	0	0	0.29	
	Badagry	36	0	0	0	0	
	Ibeju-Lekki	36	1	0	0	0.17	
	**Total**	**144**	**18**	0	0		
AChE1 (G119S)	Alimosho	36	0	23	05	0.32	0.00
	Kosofe	36	0	26	07	0.36	0.00
	Badagry	36	0	0	0	0	
	Ibeju-Lekki	36	0	0	0	0	
	**Total**						

NB: H–W is the probability of the exact test for goodness of fit to Hardy–Weinberg equilibrium; P significant at <0.05. RR: homozygote resistance RS: heterozygote resistance and SS: homozygote susceptible.

## Discussion

The WHO and SDGs aim to reduce the global malaria prevalence by 90% in the year 2030, therefore, the regular monitoring of the limited available malaria control options including the use of insecticides needs to be harnessed strategically. IRS and ITNs are the two main insecticides-based strategies in malaria vector control^2^. The efficacy of the few Pyrethriods, DDT and carbamates insecticides currently approved for use in public health is critical to the sustainability of these strategies.

In this study, high level of resistance to DDT and permethrin was recorded in *An. gambiae s.l*. from the evaluated LGAs of Lagos State. Several previous studies in different parts of Nigeria have recorded similar resistant level to different Pyrethriods and DDT^7,9,11,12^. Pyrethriods resistance recorded in these study areas could be detrimental to the effectiveness of LLINs as they are the only class of insecticide currently approved for use in LLINs. Though, the level of Pyrethriods resistance in this study area should not be allowed to affect the utilization of LLINs in these areas because it also offers partial protections to users by serving as barrier from mosquito bites thereby reducing the risk of infection^30^. Suspected resistance to bendiocarb was recorded in Alimosho and Kosofe LGAs in this study. Another previous study has also reported resistance to propoxur in several parts of Lagos State^31^. Resistance to carbamates could have a detrimental effect on malaria vector being one of the few alternative available to widely used Pyrethriods especially with the widespread resistance to DDT and Pyrethriods.

Result from this study also highlights the relevance of PBO based control measures in DDT and Pyrethriods resistance management in malaria vector in Lagos State, Nigeria. PBO synergized bioassays did not only achieve faster knockdown rate and time in DDT and permethrin resistant *An. gambiae s.s*, it also recorded significantly higher 24hrs percentage mortality. Similarly, studies in several parts of Africa have also proven the efficacy of PBO synergist plus insecticides in resistant malaria vector management^24,28,29,31–33^.

Elevated level of detoxifying enzymes (cytochrome P450, glutathione S-transferase, esterase α and esterase β) activities was observed in field population of *An. gambiae* s.s. when compared to that of the laboratory susceptible Kisumu strain in this study. Also, studies have linked the increased activities of detoxifying enzymes (P450s and GSTs) and/or mutation in some P450s and GSTs gene to Pyrethriods and other classes of neurotoxic insecticides resistance in malaria vector^7,16,34–38^. Likewise increased activities of esterase α and esterase β have been implicated in resistance to different classes of insecticides^39,40^.

The *kdr-w* (L1014F) mutation was detected in all the study LGAs in this study. Previous studies in Nigeria^8,9,15,31^ and several parts of West and Central Africa^16,41–43^ have described similar mutation to Pyrethriods and DDT which confers resistance to the vectors. Finding from this study also showed the presence of *kdr-e* (L1014S) mutation in DDT and Pyrethriods resistant *An. gambiae s.s*. for first time Nigeria. L1014S mutation had been previously characterized as the East African type of *kdr*^14^ but later study discovered that both knockdown mutation co-occur in Gabon and Uganda^18,19^. Recently however, L1014S mutation had been detected in malaria vectors from some parts of West Africa^44–47^. L1014F and L1014S mutations have been associated with DDT and Pyrethriods cross resistance in *An. gambiae*^13,14^ though it has been argued that this mutation alone may not solely be responsible for this phenotypic response^48^. Highlighting the importance of multiple resistance mechanisms in the high level of resistance reported in *An. gambiae* s.s. to DDT and Pyrethriods in this study, the allele frequencies of L1014F recorded in this study is higher than what was previously reported in Senegal^49^ but lower than that of Burkina Faso^42^. The observed genotypic frequencies of L1014F mutations from all the locations in this study did not depart significantly from the Hardy-Weinberg proportions. Previous studies from Cameroon and Burkina Faso reported varies departure from Hardy-Weinberg proportions with locations^42,50^.

The *Ace-1*^*R*^ (G119S) mutation was detected in *An. gambiae s.s*. from Alimosho and Kosofe LGAs of Lagos State, though the genotype frequency significantly departs from the Hardy-Weinberg equilibrium which maybe result from the excess heterozygotes resistant genotype in the *An. gambiae s.s*. population. Previous studies on malaria vectors in southern Nigeria have reported resistance to carbamates^10,31^ but did not detect the *Ace-1*^*R*^ mutation that has been linked to carbamates and organophosphate cross resistance in malaria vectors. The detection of *Ace-1*^*R*^ mutation in *An. gambiae* s.s. in Lagos State could be detrimental to the utilization and efficacy of IRS, a major strategy in the control of malaria vector in Sub Saharan Africa. Previous studies have associated the extensive use of agricultural pesticides^42,51,52^ and spread of resistance gene from neighbouring countries^53^ with development of carbamates and organophosphates cross resistance, but this may not be applicable in this study. The two LGAs where *Ace-1*^*R*^ mutation was detected are densely populated with little or no agricultural activities. The development of *Ace-1*^*R*^ mutation in locations can be attributed to widespread use of dichlorvos (DDVP) an organophosphate pesticide for the control of mosquitoes and other household pests^54^.

Findings from this study shows that *An. gambiae s.s*. from Lagos State exhibits multiple resistance mechanism to the different classes of insecticides available for control. It is important that regular insecticides monitoring be carried out if the WHO and SDGs goals of 2030 is to be achieved. The use PBO and other synergists incorporated into LLINs and other malaria vector control strategies should be encouraged in this areas as metabolic resistance mechanism are also proven to contribute to high level of insecticides resistance reported in the study areas.

## Materials and Methods

### Study area and sample collection

**Figure 1:**
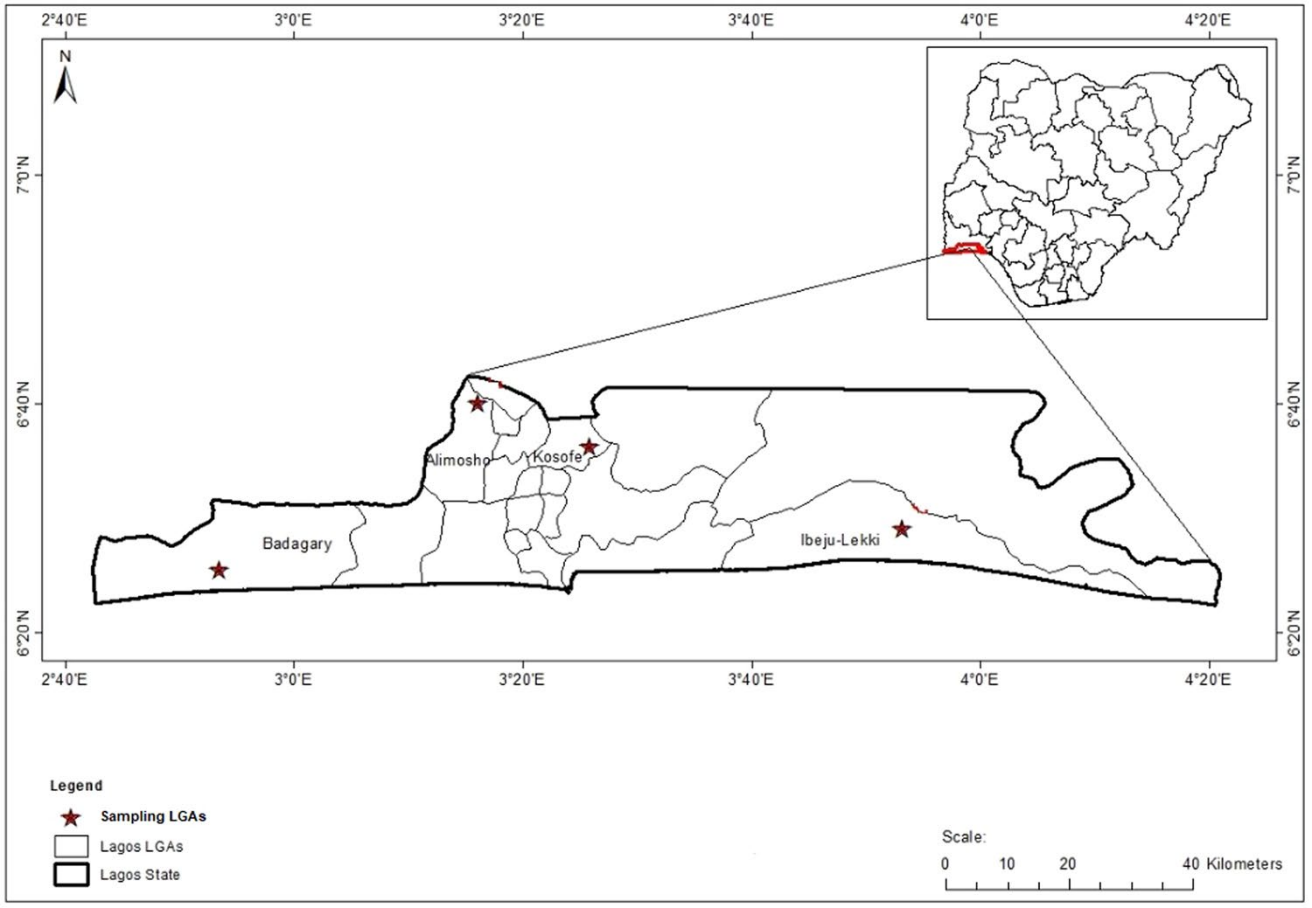
Map of Lagos State showing study LGAs where mosquito immature stages were collected. NB:Map was generated using GIS software ArcMap, version 10.1.

The study was carried out in four Local Government Areas (LGAs) of Lagos State, two densely populated LGAs: Alimosho and Kosofe and two less densely populated LGAs: Ibeju-Lekki and Badagry. According to the National Population Commission (NPC) in 2006, Badagry has an estimated population of 237,731 spanning a 443 km² area. It is the second largest town in Lagos State and surrounded by lakes, creeks and island. The major occupations known include fishing farming and salt making which is due to the abundance of trees and ocean water. Alimosho has an estimated 1,319,571 inhabitants occupying a 138 km² area of and it is the largest LGA in Lagos State. Ibeju-Lekki located in the north eastern part in the Epe Division of Lagos State, Nigeria has a total area of 455 km^2^ and a population of 117481 and Kosofe LGA with population of 66393 and an area of 81 km^2^ (Fig. 1).

**Figure 2:**
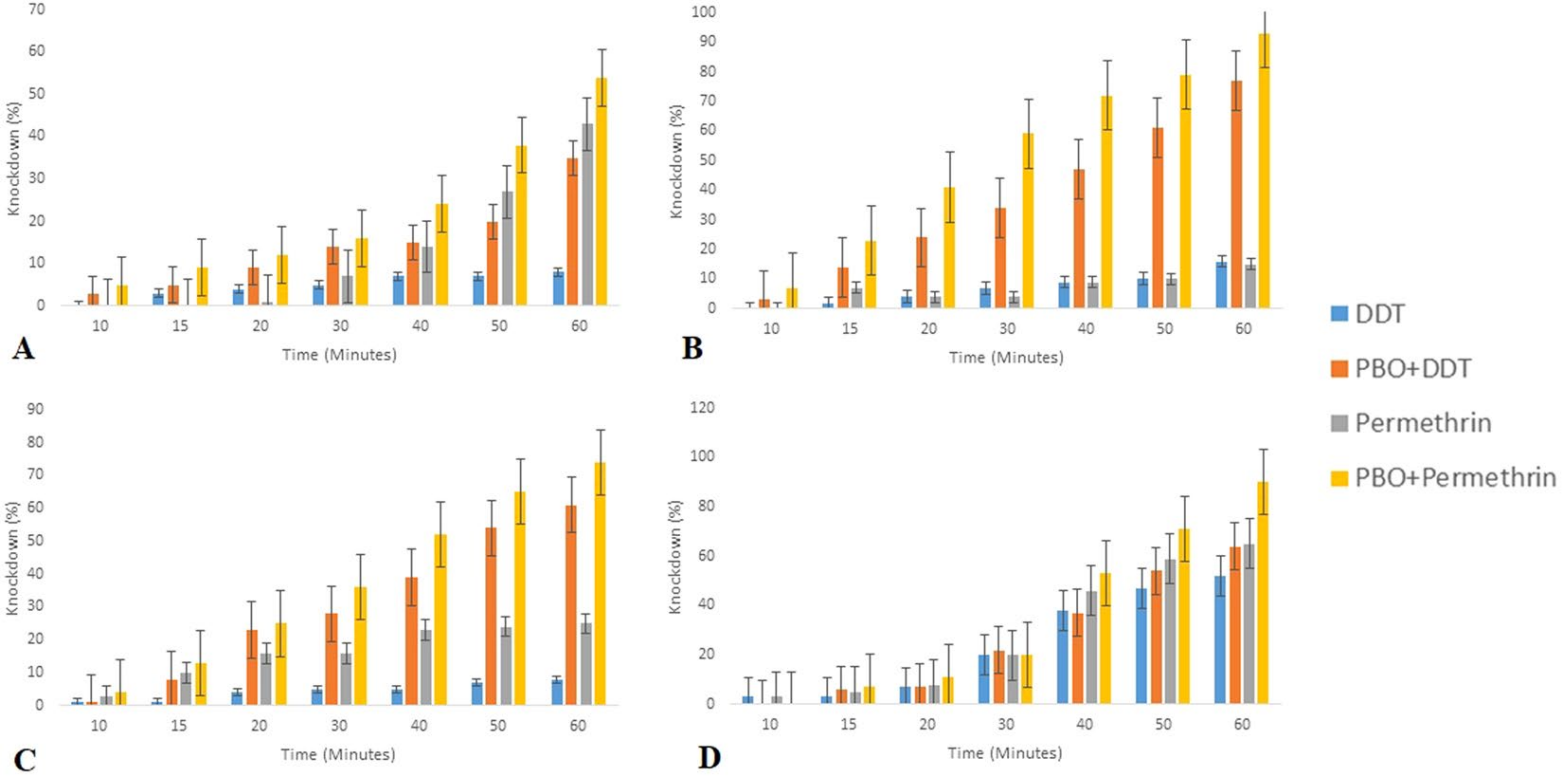
(**A**–**D**) Percentage knockdown of *An. gambiae* s.s. exposed to insecticides and PBO + insecticide from (**A**) Kosofe LGA (**B**) Ibeju lekki LGA (**C**) Alimosho LGA (**D**) Badagry LGA.

**Figure 3:**
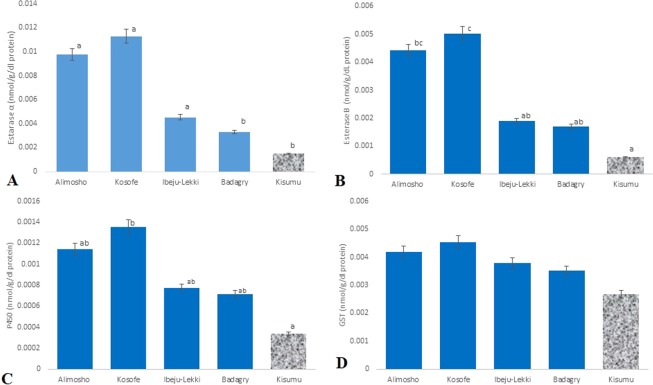
(**A**–**D**) Mean level of (**A**) cytochrome P450 (**B**) glutathione S-transferase (**C**) esterase α (**D**) esterase β activity of *Anopheles gambiae* s.l. collected in four LGAs of Lagos State, compared with the susceptible Kisumu strain (letters a,b and c used to indicate statistical differences at (P < 0.05)).

### Collection of *anopheles* mosquito

Immature stages was collected from selected sites using “dipping” technique^55^, *Anopheles* mosquito eggs, larvae and pupa were retrieved from automobile tyre tracks, small pools and puddles. Immediately after scooping, the larvae were kept in well-labeled containers and subsequently transferred to the insectary at the Nigerian Institute of Medical Research where they were allowed to emerge into adults under standard insectary conditions.

### Susceptibility and synergistic assay

The tests were performed using WHO test filter papers impregnated with the selected insecticides and piperonyl butoxide (PBO) from the Vector Control Research Unit (VCRU), University Sains Malaysia (http:/www. inreskit.usm.my). Non-blood fed, two to three days old female *Anopheles* were exposed to DDT (4%), Permethrin (0.75%) and Bendiocarb (0.1%) using the WHO standard procedure. A total of 25 female mosquitoes were pre-exposed to 4% piperonyl butoxide (PBO) and this was replicated in four places. PBO treated mosquitoes were exposed to either DDT (4%) or Permethrin (0.75%) for another one hour, each experiment consisted of four replicates. Knockdown rates of mosquitoes were recorded at intervals for one hour. Mosquitoes were later transferred into holding tubes with untreated papers; allowed a 24-hour recovery period and supplied with a 10% sugar meal during this period after which mortality was recorded. All the bioassays were accompanied by negative control.

### Mosquito identification

The genomic DNA of *Anopheles gambiae* that has been identified using morphological keys^56^ was extracted according using protocols as earlier described^57^. Further, molecular identification of mosquito samples was carried out^57^. Four primers including; ME, AR, QD, UN, GA (Table 4) were used for *Anopheles gambiae*. This was done to identify sibling species of the *An. gambiae* complex. Digestion was achieved by utilizing 0.5 μl *HhaI* restriction enzyme and 10 μl of the PCR product from the reaction above. It was incubated at 37 °C for 24 hours and the PCR fragments were resolved on a 1.5% agarose gel stained with ethidium bromide and visualized under UV light^58^.

**Table 4:** Primers used for the molecular identification and target-site modifications screening in *An. gambiae* in Lagos State, Nigeria.

	Primer name	Primer sequence 5′−3′	References
*An. gambiae* s.l. identification	ME	TGACCAACCCACTCCCTTGA	^61^
	AR	AAGTGTCCTTCTCCATCCTA	
	QD	CAGACCAAGATGGTTAGTAT	
	UN	GTGTGCCCCTTCCTCGATGT	
	GA	GTGTGCCCCTTCCTCGATGT	
L1014F -*kdr* mutation	Agd1	CTGGTTTGGTCGGCACGTTT	^13^
	Agd2	GCAAGGCTAAGAAAAGGTTAAG	
	Agd3	CCACCGTAGTGATAGGAAATTTA	
	Agd4	CCACCGTAGTGATAGGAAATTTT	
L1014S -*kdr* mutation	Agd1	GTGGAACTTCACCGACTTC	^14^
	Agd2	GCAAGGCTAAGAAAAGGTTAAG	
	Agd4	CCACCGTAGTGATAGGAAATTTT	
	Agd5	TTTGCATTACTTACGACTG	
G119S -*ace-1* mutation	Moustdir1	CGGGNGCSACYATGTGGAA	^62^
	Moustrev1	ACGATMACGTTCTCYTCCGA	

### Knockdown resistance (L1014F and L1014S) characterization

L1014F mutations assay (*kdr* west) was carried out as described by^13^ using the following primer pairs: Agd1, Agd2, Agd3 and Agd4 (Table 4). L1014S mutation assay (*kdr* east) was carried out as described by^14^. The primers Agd1, Agd2, Agd4 and Agd5 (Table 4) were used for the assay. The PCR condition for all assay includes an initial denaturation of 95 °C for 5 minutes, then 40 cycles at 95 °C for one minute, 48 °C for 2 minutes, 72 °C for 2 minutes and final extension at 72 °C for 10 minutes.

The PCR reactions were carried out in a total volume of 20 ul containing 12.5 µl of PCR master mix containing 1 × PCR buffer, 1.5 mM MgCl2, 0.2 mM of each dNTP, 0.4 µM of each primer, one unit of *Taq polymerase* (Solis BioDyne) and 1 µl of genomic DNA. 10ul of PCR product and 1ul of loading buffer was loaded into each sample well on a 1.5% agarose gel visualised by ethidium bromide stains under Ultra Violet light (UV light).

### Insensitivity acetylcholine (AChE1) assay

PCR-RFLP analysis was used for *Ace-1*^*R*^ mutation detection as described by^59^. The *Ace-1*^*R*^ SNP region was amplified using two primers MOUSTDIR1 and MOUSTREV1 (Table 4). PCR was carried out in a 25-μl reaction volume using *Taq polymerase* (Solis BioDyne). PCR reaction contains 1 μl of genomic DNA under the following amplification condition: 94 °C/2 minutes, (98 °C/10 s, 68 °C/30 s) × 35cycles, 68 °C/2 minutes. The PCR product (15 μl) was digested by adding 1 μl Alu I restriction enzyme, 2 μl of H_2_0, and 2 μl of buffer and incubated at 37 °C for 15 minutes. Digested fragments were resolved in 1.5% agarose gel and visualized under Ultra Violet light (UV light).

### Metabolic enzyme activity assay

Eight adult female mosquito that were pre-exposed to WHO standard insecticide test paper were homogenized singly in 200 μl of cold distilled water in a 1.5 mL centrifuge tube. The homogenate was centrifuged at 14,000 rpm for 20 seconds and the supernatant stored at −20 °C (Hemingway 1998). Three metabolic enzymes, Cytochrome P450 monooxygenase (P450s), Glutathione S-transferase (GSTs), and non-specific esterase (carboxylesterase) assay (COEs) (hydrolyzing α- and β- napthyl acetate) were analyzed on single individuals of field collected Insecticide resistant females mosquitoes, and on the laboratory susceptible strain according to WHO protocols (Hemingway 1998). Mean absorbance values for each tested mosquito and enzyme were converted into enzyme activity and standardized based on the total protein amount.

### Glutathione S-transferase assay

This test was carried out in two replicates. 10 µl of mosquito homogenate were placed in separate well in microtitre plate and 200 µl of the GSH (reduced glutathione)/CDNB (1-chloro-2,4′-dinitrobenzene) working solution was then added. Three plate blanks containing 10 µl distill water and 200 µl of the GSH/CDNB working solution were used per microtitre plate as negative control. The test was then left at room temperature for 20 minutes and the absorbance value was read at 340 nm at end.

### Cytochrome P450 monooxygenase assay

Mosquito homogenate (2 µl) of were placed in separate wells of the microplate, 80 µl of 0.625 M potassium phosphate (pH 7.2) was added to each replicate. Then 200 µl of the mixture of 5 ml methanol solution of tetramethyl benzidine with 15 ml of 0.25 M sodium acetate buffer (pH 5.0) was added to each well, 25 µl of 3% hydrogen peroxide was also added to each replicate, the preparation was left for 2 hours at room temperature before reading of absorbance at 650 nm. Control was run at 20 µl of buffer instead of mosquito homogenate and the assay was carried out in duplicate.

### Esterase α and β assay

20 µl of mosquito homogenate were placed in separate wells of microtitre plates in two replicates, 200 µl of 1-NA (naphthyl acetate) working solution was added to one replicate and 200 µl of 2-NA was added to the second replicate and left at room temperature for 15 minutes, after which 50 µl of fast blue stain solution was added. Three plate blank solutions were prepared per plate and blank wells contain 20 µl of distill water, 200 µl of 1-NA or 2-NA solution and 50 µl of stain. The plates were rear at 570 nm wavelength.

### Protein assay

Total protein was measured for each mosquito using Biuret test. All measurements were done in duplicate. Protein concentration in sample was calculated as absorbance of sample/absorbance of standard multiply by concentration of standard (60 g/dl). Enzyme activities were calculated as sample absorbance/g/dl of protein.

### Statistical analysis

Susceptibility/resistance to test insecticides was categorized based on the 98–100% mortality criteria of mosquito which implies susceptibility, 80–97% mortality indicates suspected resistance that needs further confirmation through biochemical or molecular assays and <80% mortality implies resistance^60^. Regression probit was used to compute the KDT_50_ and KDT_95_. Chi-square was used to compare percentage mortality between insecticide only, and PBO plus insecticide. Analysis of variance (ANOVA) was used to determine the difference in the activities of detoxifying enzymes in wild and laboratory susceptible strain of *Anopheles* mosquito and Duncan multiple range test was check for statistical similarities/differences between the locations. The frequency of *kdr-w. kdr-e and Ace-1*^*R*^ mutations in *An. gambiae* s.s. population were compared to Hardy-Weinberg expectations using Pearson’s chi-square test. All data analyses were computed using Microsoft Excel version 2016 and IBM SPSS Statistics 23. P-value of <0.05 was considered statistically significant.

## Supplementary information

Supplementary Information.

Supplementary Information 2.

Supplementary Information 3.
