# Supplementary material for: First report of AChE1 (G119S) mutation and multiple resistance mechanisms in Anopheles gambiae s.s. in Nigeria
Source: Sci Rep. 2020 May 4;10:7482. doi: 10.1038/s41598-020-64412-7 (PMC7198501; doi:10.1038/s41598-020-64412-7)
Supplement: Supplementary file 1 — Supplementary Information. [file 41598_2020_64412_MOESM1_ESM.doc]

Between-Subjects Factors	
	Value Label	N	
Location	1	Kosofe	8	
	2	Alimosho	8	
	3	Ibeju-Lekki	8	
	4	Badagry	8	
	5	Kisumu	6	


Descriptive Statistics	
	Location	Mean	Std. Deviation	N	
Esterase á	Kosofe	.01128798	.006527450	8	
	Alimosho	.00978973	.006369571	8	
	Ibeju-Lekki	.00451543	.001613612	8	
	Badagry	.00329397	.001365436	8	
	Kisumu	.00148694	.000491505	6	
	Total	.00631628	.005570081	38	
Esterase â	Kosofe	.00501711	.003702923	8	
	Alimosho	.00442480	.003809297	8	
	Ibeju-Lekki	.00189112	.000760810	8	
	Badagry	.00170200	.000684729	8	
	Kisumu	.00060089	.000196101	6	
	Total	.00283910	.002897992	38	
Cytochrome P450	Kosofe	.00135449	.001093871	8	
	Alimosho	.00114430	.001136828	8	
	Ibeju-Lekki	.00077268	.000236273	8	
	Badagry	.00071086	.000217371	8	
	Kisumu	.00033435	.000086070	6	
	Total	.00089118	.000782300	38	
GST	Kosofe	.00454225	.003190285	8	
	Alimosho	.00419645	.003376463	8	
	Ibeju-Lekki	.00377826	.001953940	8	
	Badagry	.00351379	.001817164	8	
	Kisumu	.00266945	.000664288	6	
	Total	.00379639	.002422237	38	


Multivariate Testsa	
Effect	Value	F	Hypothesis df	Error df	Sig.	
Intercept	Pillai's Trace	.860	46.089b	4.000	30.000	.000	
	Wilks' Lambda	.140	46.089b	4.000	30.000	.000	
	Hotelling's Trace	6.145	46.089b	4.000	30.000	.000	
	Roy's Largest Root	6.145	46.089b	4.000	30.000	.000	
Location	Pillai's Trace	.871	2.297	16.000	132.000	.005	
	Wilks' Lambda	.217	3.747	16.000	92.289	.000	
	Hotelling's Trace	3.216	5.728	16.000	114.000	.000	
	Roy's Largest Root	3.090	25.490c	4.000	33.000	.000	

a. Design: Intercept + Location	
b. Exact statistic	
c. The statistic is an upper bound on F that yields a lower bound on the significance level.	


Tests of Between-Subjects Effects	
Source	Dependent Variable	Type III Sum of Squares	df	Mean Square	F		
Corrected Model	Esterase á	.001a	4	.000	7.156		
	Esterase â	.000b	4	2.641E-5	4.250		
	Cytochrome P450	4.463E-6c	4	1.116E-6	2.025		
	GST	1.399E-5d	4	3.498E-6	.568		
Intercept	Esterase á	.001	1	.001	74.288		
	Esterase â	.000	1	.000	44.879		
	Cytochrome P450	2.795E-5	1	2.795E-5	50.732		
	GST	.001	1	.001	85.231		
Location	Esterase á	.001	4	.000	7.156		
	Esterase â	.000	4	2.641E-5	4.250		
	Cytochrome P450	4.463E-6	4	1.116E-6	2.025		
	GST	1.399E-5	4	3.498E-6	.568		
Error	Esterase á	.001	33	1.863E-5			
	Esterase â	.000	33	6.215E-6			
	Cytochrome P450	1.818E-5	33	5.509E-7			
	GST	.000	33	6.154E-6			
Total	Esterase á	.003	38				
	Esterase â	.001	38				
	Cytochrome P450	5.282E-5	38				
	GST	.001	38				
Corrected Total	Esterase á	.001	37				
	Esterase â	.000	37				
	Cytochrome P450	2.264E-5	37				
	GST	.000	37				

Tests of Between-Subjects Effects	
Source	Dependent Variable	Sig.	
Corrected Model	Esterase á	.000	
	Esterase â	.007	
	Cytochrome P450	.114	
	GST	.687	
Intercept	Esterase á	.000	
	Esterase â	.000	
	Cytochrome P450	.000	
	GST	.000	
Location	Esterase á	.000	
	Esterase â	.007	
	Cytochrome P450	.114	
	GST	.687	
Error	Esterase á		
	Esterase â		
	Cytochrome P450		
	GST		
Total	Esterase á		
	Esterase â		
	Cytochrome P450		
	GST		
Corrected Total	Esterase á		
	Esterase â		
	Cytochrome P450		
	GST		

a. R Squared = .464 (Adjusted R Squared = .400)	
b. R Squared = .340 (Adjusted R Squared = .260)	
c. R Squared = .197 (Adjusted R Squared = .100)	
d. R Squared = .064 (Adjusted R Squared = -.049)	


Estimated Marginal Means


Location


Estimates	
Dependent Variable	Location	Mean	Std. Error	95% Confidence Interval	
				Lower Bound	Upper Bound	
Esterase á	Kosofe	.011	.002	.008	.014	
	Alimosho	.010	.002	.007	.013	
	Ibeju-Lekki	.005	.002	.001	.008	
	Badagry	.003	.002	.000	.006	
	Kisumu	.001	.002	-.002	.005	
Esterase â	Kosofe	.005	.001	.003	.007	
	Alimosho	.004	.001	.003	.006	
	Ibeju-Lekki	.002	.001	9.794E-5	.004	
	Badagry	.002	.001	-9.117E-5	.003	
	Kisumu	.001	.001	-.001	.003	
Cytochrome P450	Kosofe	.001	.000	.001	.002	
	Alimosho	.001	.000	.001	.002	
	Ibeju-Lekki	.001	.000	.000	.001	
	Badagry	.001	.000	.000	.001	
	Kisumu	.000	.000	.000	.001	
GST	Kosofe	.005	.001	.003	.006	
	Alimosho	.004	.001	.002	.006	
	Ibeju-Lekki	.004	.001	.002	.006	
	Badagry	.004	.001	.002	.005	
	Kisumu	.003	.001	.001	.005	


Pairwise Comparisons	
Dependent Variable	(I) Location	(J) Location	Mean Difference (I-J)	Std. Error	Sig.b	95% Confidence Interval for Differenceb	
						Lower Bound	Upper Bound	
Esterase á	Kosofe	Alimosho	.001	.002	.492	-.003	.006	
		Ibeju-Lekki	.007*	.002	.004	.002	.011	
		Badagry	.008*	.002	.001	.004	.012	
		Kisumu	.010*	.002	.000	.005	.015	
	Alimosho	Kosofe	-.001	.002	.492	-.006	.003	
		Ibeju-Lekki	.005*	.002	.020	.001	.010	
		Badagry	.006*	.002	.005	.002	.011	
		Kisumu	.008*	.002	.001	.004	.013	
	Ibeju-Lekki	Kosofe	-.007*	.002	.004	-.011	-.002	
		Alimosho	-.005*	.002	.020	-.010	-.001	
		Badagry	.001	.002	.575	-.003	.006	
		Kisumu	.003	.002	.203	-.002	.008	
	Badagry	Kosofe	-.008*	.002	.001	-.012	-.004	
		Alimosho	-.006*	.002	.005	-.011	-.002	
		Ibeju-Lekki	-.001	.002	.575	-.006	.003	
		Kisumu	.002	.002	.444	-.003	.007	
	Kisumu	Kosofe	-.010*	.002	.000	-.015	-.005	
		Alimosho	-.008*	.002	.001	-.013	-.004	
		Ibeju-Lekki	-.003	.002	.203	-.008	.002	
		Badagry	-.002	.002	.444	-.007	.003	
Esterase â	Kosofe	Alimosho	.001	.001	.638	-.002	.003	
		Ibeju-Lekki	.003*	.001	.017	.001	.006	
		Badagry	.003*	.001	.012	.001	.006	
		Kisumu	.004*	.001	.002	.002	.007	
	Alimosho	Kosofe	-.001	.001	.638	-.003	.002	
		Ibeju-Lekki	.003	.001	.050	-2.251E-6	.005	
		Badagry	.003*	.001	.036	.000	.005	
		Kisumu	.004*	.001	.008	.001	.007	
	Ibeju-Lekki	Kosofe	-.003*	.001	.017	-.006	-.001	
		Alimosho	-.003	.001	.050	-.005	2.251E-6	
		Badagry	.000	.001	.880	-.002	.003	
		Kisumu	.001	.001	.345	-.001	.004	
	Badagry	Kosofe	-.003*	.001	.012	-.006	-.001	
		Alimosho	-.003*	.001	.036	-.005	.000	
		Ibeju-Lekki	.000	.001	.880	-.003	.002	
		Kisumu	.001	.001	.419	-.002	.004	
	Kisumu	Kosofe	-.004*	.001	.002	-.007	-.002	
		Alimosho	-.004*	.001	.008	-.007	-.001	
		Ibeju-Lekki	-.001	.001	.345	-.004	.001	
		Badagry	-.001	.001	.419	-.004	.002	
Cytochrome P450	Kosofe	Alimosho	.000	.000	.575	-.001	.001	
		Ibeju-Lekki	.001	.000	.126	.000	.001	
		Badagry	.001	.000	.092	.000	.001	
		Kisumu	.001*	.000	.016	.000	.002	
	Alimosho	Kosofe	.000	.000	.575	-.001	.001	
		Ibeju-Lekki	.000	.000	.324	.000	.001	
		Badagry	.000	.000	.251	.000	.001	
		Kisumu	.001	.000	.052	-5.606E-6	.002	
	Ibeju-Lekki	Kosofe	-.001	.000	.126	-.001	.000	
		Alimosho	.000	.000	.324	-.001	.000	
		Badagry	6.181E-5	.000	.869	-.001	.001	
		Kisumu	.000	.000	.282	.000	.001	
	Badagry	Kosofe	-.001	.000	.092	-.001	.000	
		Alimosho	.000	.000	.251	-.001	.000	
		Ibeju-Lekki	-6.181E-5	.000	.869	-.001	.001	
		Kisumu	.000	.000	.354	.000	.001	
	Kisumu	Kosofe	-.001*	.000	.016	-.002	.000	
		Alimosho	-.001	.000	.052	-.002	5.606E-6	
		Ibeju-Lekki	.000	.000	.282	-.001	.000	
		Badagry	.000	.000	.354	-.001	.000	
GST	Kosofe	Alimosho	.000	.001	.782	-.002	.003	
		Ibeju-Lekki	.001	.001	.542	-.002	.003	
		Badagry	.001	.001	.413	-.001	.004	
		Kisumu	.002	.001	.171	-.001	.005	
	Alimosho	Kosofe	.000	.001	.782	-.003	.002	
		Ibeju-Lekki	.000	.001	.738	-.002	.003	
		Badagry	.001	.001	.586	-.002	.003	
		Kisumu	.002	.001	.263	-.001	.004	
	Ibeju-Lekki	Kosofe	-.001	.001	.542	-.003	.002	
		Alimosho	.000	.001	.738	-.003	.002	
		Badagry	.000	.001	.832	-.002	.003	
		Kisumu	.001	.001	.414	-.002	.004	
	Badagry	Kosofe	-.001	.001	.413	-.004	.001	
		Alimosho	-.001	.001	.586	-.003	.002	
		Ibeju-Lekki	.000	.001	.832	-.003	.002	
		Kisumu	.001	.001	.533	-.002	.004	
	Kisumu	Kosofe	-.002	.001	.171	-.005	.001	
		Alimosho	-.002	.001	.263	-.004	.001	
		Ibeju-Lekki	-.001	.001	.414	-.004	.002	
		Badagry	-.001	.001	.533	-.004	.002	

Based on estimated marginal means	
*. The mean difference is significant at the .05 level.	
b. Adjustment for multiple comparisons: Least Significant Difference (equivalent to no adjustments).	


Multivariate Tests	
	Value	F	Hypothesis df	Error df	Sig.	
Pillai's trace	.871	2.297	16.000	132.000	.005	
Wilks' lambda	.217	3.747	16.000	92.289	.000	
Hotelling's trace	3.216	5.728	16.000	114.000	.000	
Roy's largest root	3.090	25.490a	4.000	33.000	.000	

Each F tests the multivariate effect of Location. These tests are based on the linearly independent pairwise comparisons among the estimated marginal means.	
a. The statistic is an upper bound on F that yields a lower bound on the significance level.	


Univariate Tests	
Dependent Variable	Sum of Squares	df	Mean Square	F	Sig.	
Esterase á	Contrast	.001	4	.000	7.156	.000	
	Error	.001	33	1.863E-5			
Esterase â	Contrast	.000	4	2.641E-5	4.250	.007	
	Error	.000	33	6.215E-6			
Cytochrome P450	Contrast	4.463E-6	4	1.116E-6	2.025	.114	
	Error	1.818E-5	33	5.509E-7			
GST	Contrast	1.399E-5	4	3.498E-6	.568	.687	
	Error	.000	33	6.154E-6			

The F tests the effect of Location. This test is based on the linearly independent pairwise comparisons among the estimated marginal means.	


Post Hoc Tests


Location


Multiple Comparisons	
Dependent Variable	(I) Location	(J) Location	Mean Difference (I-J)	Std. Error	Sig.	95% Confidence Interval	
						Lower Bound	Upper Bound	
Esterase á	Tamhane	Kosofe	Alimosho	.00149825	.003224497	1.000	-.00918937	.01218587	
			Ibeju-Lekki	.00677256	.002377271	.199	-.00235890	.01590401	
			Badagry	.00799402	.002357754	.098	-.00116413	.01715217	
			Kisumu	.00980104*	.002316509	.037	.00056452	.01903756	
		Alimosho	Kosofe	-.00149825	.003224497	1.000	-.01218587	.00918937	
			Ibeju-Lekki	.00527431	.002323122	.422	-.00363232	.01418093	
			Badagry	.00649577	.002303146	.212	-.00243723	.01542876	
			Kisumu	.00830279	.002260905	.075	-.00070937	.01731495	
		Ibeju-Lekki	Kosofe	-.00677256	.002377271	.199	-.01590401	.00235890	
			Alimosho	-.00527431	.002323122	.422	-.01418093	.00363232	
			Badagry	.00122146	.000747342	.737	-.00126758	.00371050	
			Kisumu	.00302848*	.000604757	.008	.00077925	.00527772	
		Badagry	Kosofe	-.00799402	.002357754	.098	-.01715217	.00116413	
			Alimosho	-.00649577	.002303146	.212	-.01542876	.00243723	
			Ibeju-Lekki	-.00122146	.000747342	.737	-.00371050	.00126758	
			Kisumu	.00180702	.000522795	.067	-.00009926	.00371331	
		Kisumu	Kosofe	-.00980104*	.002316509	.037	-.01903756	-.00056452	
			Alimosho	-.00830279	.002260905	.075	-.01731495	.00070937	
			Ibeju-Lekki	-.00302848*	.000604757	.008	-.00527772	-.00077925	
			Badagry	-.00180702	.000522795	.067	-.00371331	.00009926	
Esterase â	Tamhane	Kosofe	Alimosho	.00059231	.001878243	1.000	-.00563337	.00681799	
			Ibeju-Lekki	.00312600	.001336529	.396	-.00207081	.00832281	
			Badagry	.00331511	.001331376	.332	-.00189013	.00852035	
			Kisumu	.00441622	.001311627	.112	-.00082930	.00966175	
		Alimosho	Kosofe	-.00059231	.001878243	1.000	-.00681799	.00563337	
			Ibeju-Lekki	.00253369	.001373389	.668	-.00281483	.00788221	
			Badagry	.00272280	.001368375	.586	-.00263413	.00807973	
			Kisumu	.00382391	.001349167	.224	-.00157263	.00922046	
		Ibeju-Lekki	Kosofe	-.00312600	.001336529	.396	-.00832281	.00207081	
			Alimosho	-.00253369	.001373389	.668	-.00788221	.00281483	
			Badagry	.00018911	.000361885	1.000	-.00101261	.00139083	
			Kisumu	.00129022*	.000280648	.016	.00022831	.00235214	
		Badagry	Kosofe	-.00331511	.001331376	.332	-.00852035	.00189013	
			Alimosho	-.00272280	.001368375	.586	-.00807973	.00263413	
			Ibeju-Lekki	-.00018911	.000361885	1.000	-.00139083	.00101261	
			Kisumu	.00110111*	.000254982	.022	.00014641	.00205582	
		Kisumu	Kosofe	-.00441622	.001311627	.112	-.00966175	.00082930	
			Alimosho	-.00382391	.001349167	.224	-.00922046	.00157263	
			Ibeju-Lekki	-.00129022*	.000280648	.016	-.00235214	-.00022831	
			Badagry	-.00110111*	.000254982	.022	-.00205582	-.00014641	
Cytochrome P450	Tamhane	Kosofe	Alimosho	.00021018	.000557778	1.000	-.00163887	.00205924	
			Ibeju-Lekki	.00058181	.000395661	.865	-.00095209	.00211571	
			Badagry	.00064362	.000394304	.788	-.00089237	.00217962	
			Kisumu	.00102014	.000388335	.289	-.00052741	.00256770	
		Alimosho	Kosofe	-.00021018	.000557778	1.000	-.00205924	.00163887	
			Ibeju-Lekki	.00037163	.000410519	.993	-.00122354	.00196679	
			Badagry	.00043344	.000409211	.980	-.00116382	.00203070	
			Kisumu	.00080996	.000403463	.584	-.00079865	.00241856	
		Ibeju-Lekki	Kosofe	-.00058181	.000395661	.865	-.00211571	.00095209	
			Alimosho	-.00037163	.000410519	.993	-.00196679	.00122354	
			Badagry	.00006181	.000113509	1.000	-.00031484	.00043847	
			Kisumu	.00043833*	.000090624	.008	.00010838	.00076829	
		Badagry	Kosofe	-.00064362	.000394304	.788	-.00217962	.00089237	
			Alimosho	-.00043344	.000409211	.980	-.00203070	.00116382	
			Ibeju-Lekki	-.00006181	.000113509	1.000	-.00043847	.00031484	
			Kisumu	.00037652*	.000084504	.013	.00007207	.00068097	
		Kisumu	Kosofe	-.00102014	.000388335	.289	-.00256770	.00052741	
			Alimosho	-.00080996	.000403463	.584	-.00241856	.00079865	
			Ibeju-Lekki	-.00043833*	.000090624	.008	-.00076829	-.00010838	
			Badagry	-.00037652*	.000084504	.013	-.00068097	-.00007207	
GST	Tamhane	Kosofe	Alimosho	.00034580	.001642347	1.000	-.00510032	.00579193	
			Ibeju-Lekki	.00076399	.001322677	1.000	-.00378751	.00531548	
			Badagry	.00102847	.001298076	.997	-.00348294	.00553987	
			Kisumu	.00187281	.001160080	.794	-.00259453	.00634015	
		Alimosho	Kosofe	-.00034580	.001642347	1.000	-.00579193	.00510032	
			Ibeju-Lekki	.00041818	.001379238	1.000	-.00436472	.00520109	
			Badagry	.00068266	.001355663	1.000	-.00406693	.00543225	
			Kisumu	.00152700	.001224177	.943	-.00320539	.00625940	
		Ibeju-Lekki	Kosofe	-.00076399	.001322677	1.000	-.00531548	.00378751	
			Alimosho	-.00041818	.001379238	1.000	-.00520109	.00436472	
			Badagry	.00026448	.000943396	1.000	-.00286503	.00339399	
			Kisumu	.00110882	.000742147	.843	-.00161631	.00383395	
		Badagry	Kosofe	-.00102847	.001298076	.997	-.00553987	.00348294	
			Alimosho	-.00068266	.001355663	1.000	-.00543225	.00406693	
			Ibeju-Lekki	-.00026448	.000943396	1.000	-.00339399	.00286503	
			Kisumu	.00084434	.000697357	.948	-.00169356	.00338225	
		Kisumu	Kosofe	-.00187281	.001160080	.794	-.00634015	.00259453	
			Alimosho	-.00152700	.001224177	.943	-.00625940	.00320539	
			Ibeju-Lekki	-.00110882	.000742147	.843	-.00383395	.00161631	
			Badagry	-.00084434	.000697357	.948	-.00338225	.00169356	

Based on observed means.
 The error term is Mean Square(Error) = 6.15E-006.	
*. The mean difference is significant at the .05 level.	


Homogeneous Subsets


Esterase á	
	Location	N	Subset	
			1	2	
Duncana,b,c	Kisumu	6	.00148694		
	Badagry	8	.00329397		
	Ibeju-Lekki	8	.00451543		
	Alimosho	8		.00978973	
	Kosofe	8		.01128798	
	Sig.		.209	.506	

Means for groups in homogeneous subsets are displayed.
 Based on observed means.
 The error term is Mean Square(Error) = 1.86E-005.	
a. Uses Harmonic Mean Sample Size = 7.500.	
b. The group sizes are unequal. The harmonic mean of the group sizes is used. Type I error levels are not guaranteed.	
c. Alpha = .05.	


Esterase â	
	Location	N	Subset	
			1	2	3	
Duncana,b,c	Kisumu	6	.00060089			
	Badagry	8	.00170200	.00170200		
	Ibeju-Lekki	8	.00189112	.00189112		
	Alimosho	8		.00442480	.00442480	
	Kosofe	8			.00501711	
	Sig.		.352	.052	.648	

Means for groups in homogeneous subsets are displayed.
 Based on observed means.
 The error term is Mean Square(Error) = 6.21E-006.	
a. Uses Harmonic Mean Sample Size = 7.500.	
b. The group sizes are unequal. The harmonic mean of the group sizes is used. Type I error levels are not guaranteed.	
c. Alpha = .05.	


Cytochrome P450	
	Location	N	Subset	
			1	2	
Duncana,b,c	Kisumu	6	.00033435		
	Badagry	8	.00071086	.00071086	
	Ibeju-Lekki	8	.00077268	.00077268	
	Alimosho	8	.00114430	.00114430	
	Kosofe	8		.00135449	
	Sig.		.060	.134	

Means for groups in homogeneous subsets are displayed.
 Based on observed means.
 The error term is Mean Square(Error) = 5.51E-007.	
a. Uses Harmonic Mean Sample Size = 7.500.	
b. The group sizes are unequal. The harmonic mean of the group sizes is used. Type I error levels are not guaranteed.	
c. Alpha = .05.	


GST	
	Location	N	Subset	
			1	
Duncana,b,c	Kisumu	6	.00266945	
	Badagry	8	.00351379	
	Ibeju-Lekki	8	.00377826	
	Alimosho	8	.00419645	
	Kosofe	8	.00454225	
	Sig.		.202	

Means for groups in homogeneous subsets are displayed.
 Based on observed means.
 The error term is Mean Square(Error) = 6.15E-006.	
a. Uses Harmonic Mean Sample Size = 7.500.	
b. The group sizes are unequal. The harmonic mean of the group sizes is used. Type I error levels are not guaranteed.	
c. Alpha = .05.	
