# Supplementary material for: First report of AChE1 (G119S) mutation and multiple resistance mechanisms in Anopheles gambiae s.s. in Nigeria
Source: Sci Rep. 2020 May 4;10:7482. doi: 10.1038/s41598-020-64412-7 (PMC7198501; doi:10.1038/s41598-020-64412-7)
Supplement: Supplementary file 2 — Supplementary Information 2. [file 41598_2020_64412_MOESM2_ESM.doc]

Kosofe DDT
Confidence Limits	
	Probability	95% Confidence Limits for Time	95% Confidence Limits for log(Time)a	
		Estimate	Lower Bound	Upper Bound	Estimate	Lower Bound	Upper Bound	
PROBIT	.010	7.951	.629	14.829	.900	-.201	1.171	
	.020	13.911	2.748	21.627	1.143	.439	1.335	
	.030	19.839	6.803	28.269	1.298	.833	1.451	
	.040	25.910	12.853	36.202	1.413	1.109	1.559	
	.050	32.195	20.040	47.638	1.508	1.302	1.678	
	.060	38.732	26.846	65.558	1.588	1.429	1.817	
	.070	45.548	32.608	92.282	1.658	1.513	1.965	
	.080	52.662	37.570	129.465	1.721	1.575	2.112	
	.090	60.092	42.063	178.970	1.779	1.624	2.253	
	.100	67.855	46.285	243.126	1.832	1.665	2.386	
	.150	112.212	66.053	900.041	2.050	1.820	2.954	
	.200	167.365	85.918	2597.728	2.224	1.934	3.415	
	.250	235.841	107.063	6485.242	2.373	2.030	3.812	
	.300	320.911	130.139	14783.848	2.506	2.114	4.170	
	.350	426.913	155.738	31766.150	2.630	2.192	4.502	
	.400	559.705	184.520	65693.026	2.748	2.266	4.818	
	.450	727.374	217.300	132758.441	2.862	2.337	5.123	
	.500	941.353	255.137	265422.171	2.974	2.407	5.424	
	.550	1218.281	299.468	530822.987	3.086	2.476	5.725	
	.600	1583.238	352.319	1073801.902	3.200	2.547	6.031	
	.650	2075.709	416.673	2224690.463	3.317	2.620	6.347	
	.700	2761.343	497.154	4794426.007	3.441	2.696	6.681	
	.750	3757.389	601.417	10982001.143	3.575	2.779	7.041	
	.800	5294.693	743.307	27642566.698	3.724	2.871	7.442	
	.850	7897.062	951.283	81088620.962	3.897	2.978	7.909	
	.900	13059.458	1297.216	314150867.734	4.116	3.113	8.497	
	.910	14746.527	1398.081	435728376.327	4.169	3.146	8.639	
	.920	16827.180	1516.545	621683762.114	4.226	3.181	8.794	
	.930	19455.370	1658.396	918958875.636	4.289	3.220	8.963	
	.940	22878.738	1832.531	1421875095.408	4.359	3.263	9.153	
	.950	27524.229	2053.517	2339217392.509	4.440	3.312	9.369	
	.960	34200.896	2347.367	4198552054.470	4.534	3.371	9.623	
	.970	44667.821	2766.761	8618083374.867	4.650	3.442	9.935	
	.980	63700.051	3442.313	22417979755.774	4.804	3.537	10.351	
	.990	111452.907	4856.757	101163823085.671	5.047	3.686	11.005	

a. Logarithm base = 10.	


Kosofe Bendiocarb


Confidence Limits	
	Probability	95% Confidence Limits for Time	95% Confidence Limits for log(Time)b	
		Estimate	Lower Bound	Upper Bound	Estimate	Lower Bound	Upper Bound	
PROBITa	.010	13.977	9.797	17.296	1.145	.991	1.238	
	.020	15.463	11.226	18.774	1.189	1.050	1.274	
	.030	16.488	12.235	19.783	1.217	1.088	1.296	
	.040	17.303	13.051	20.582	1.238	1.116	1.313	
	.050	17.996	13.752	21.258	1.255	1.138	1.328	
	.060	18.607	14.378	21.854	1.270	1.158	1.340	
	.070	19.160	14.948	22.392	1.282	1.175	1.350	
	.080	19.669	15.476	22.887	1.294	1.190	1.360	
	.090	20.144	15.971	23.348	1.304	1.203	1.368	
	.100	20.591	16.440	23.783	1.314	1.216	1.376	
	.150	22.551	18.515	25.694	1.353	1.268	1.410	
	.200	24.241	20.323	27.357	1.385	1.308	1.437	
	.250	25.791	21.987	28.906	1.411	1.342	1.461	
	.300	27.267	23.568	30.409	1.436	1.372	1.483	
	.350	28.711	25.100	31.914	1.458	1.400	1.504	
	.400	30.151	26.609	33.458	1.479	1.425	1.524	
	.450	31.613	28.113	35.075	1.500	1.449	1.545	
	.500	33.121	29.629	36.801	1.520	1.472	1.566	
	.550	34.702	31.175	38.677	1.540	1.494	1.587	
	.600	36.385	32.770	40.753	1.561	1.515	1.610	
	.650	38.210	34.443	43.092	1.582	1.537	1.634	
	.700	40.232	36.233	45.786	1.605	1.559	1.661	
	.750	42.535	38.199	48.972	1.629	1.582	1.690	
	.800	45.256	40.438	52.881	1.656	1.607	1.723	
	.850	48.646	43.130	57.946	1.687	1.635	1.763	
	.900	53.276	46.667	65.160	1.727	1.669	1.814	
	.910	54.459	47.550	67.052	1.736	1.677	1.826	
	.920	55.773	48.524	69.178	1.746	1.686	1.840	
	.930	57.256	49.611	71.601	1.758	1.696	1.855	
	.940	58.958	50.848	74.418	1.771	1.706	1.872	
	.950	60.961	52.288	77.778	1.785	1.718	1.891	
	.960	63.401	54.023	81.934	1.802	1.733	1.913	
	.970	66.535	56.222	87.369	1.823	1.750	1.941	
	.980	70.943	59.265	95.187	1.851	1.773	1.979	
	.990	78.491	64.359	109.019	1.895	1.809	2.038	

a. A heterogeneity factor is used.	
b. Logarithm base = 10.	


Alimosho DDT


Probit Analysis


Confidence Limits	
	Probability	95% Confidence Limits for Time	95% Confidence Limits for log(Time)a	
		Estimate	Lower Bound	Upper Bound	Estimate	Lower Bound	Upper Bound	
PROBIT	.010	8.872	.734	16.101	.948	-.134	1.207	
	.020	15.429	3.290	23.489	1.188	.517	1.371	
	.030	21.917	8.207	30.998	1.341	.914	1.491	
	.040	28.541	15.312	40.718	1.455	1.185	1.610	
	.050	35.380	23.085	55.992	1.549	1.363	1.748	
	.060	42.478	29.867	80.498	1.628	1.475	1.906	
	.070	49.864	35.494	116.717	1.698	1.550	2.067	
	.080	57.560	40.404	166.899	1.760	1.606	2.222	
	.090	65.587	44.913	233.856	1.817	1.652	2.369	
	.100	73.961	49.191	321.050	1.869	1.692	2.507	
	.150	121.638	69.409	1231.494	2.085	1.841	3.090	
	.200	180.632	89.757	3644.677	2.257	1.953	3.562	
	.250	253.582	111.359	9290.968	2.404	2.047	3.968	
	.300	343.890	134.859	21576.028	2.536	2.130	4.334	
	.350	456.054	160.848	47159.008	2.659	2.206	4.674	
	.400	596.140	189.981	99113.747	2.775	2.279	4.996	
	.450	772.504	223.065	203446.433	2.888	2.348	5.308	
	.500	996.941	261.142	413030.852	2.999	2.417	5.616	
	.550	1286.582	305.627	838777.015	3.109	2.485	5.924	
	.600	1667.210	358.508	1723357.622	3.222	2.554	6.236	
	.650	2179.328	422.708	3628264.222	3.338	2.626	6.560	
	.700	2890.143	502.747	7952749.066	3.461	2.701	6.901	
	.750	3919.408	606.095	18552112.427	3.593	2.783	7.268	
	.800	5502.297	746.229	47656299.169	3.741	2.873	7.678	
	.850	8170.856	950.775	143150488.148	3.912	2.978	8.156	
	.900	13438.013	1289.278	571370860.031	4.128	3.110	8.757	
	.910	15153.812	1387.647	798219938.279	4.181	3.142	8.902	
	.920	17266.957	1503.016	1147818326.048	4.237	3.177	9.060	
	.930	19932.134	1640.948	1711333801.494	4.300	3.215	9.233	
	.940	23397.830	1809.981	2673446369.294	4.369	3.258	9.427	
	.950	28091.802	2024.078	4446702063.201	4.449	3.306	9.648	
	.960	34823.232	2308.128	8084558027.618	4.542	3.363	9.908	
	.970	45347.844	2712.436	16859267129.808	4.657	3.433	10.227	
	.980	64418.971	3361.416	44787678207.098	4.809	3.527	10.651	
	.990	112022.458	4713.178	208921767002.664	5.049	3.673	11.320	

a. Logarithm base = 10.	


Alimosho Permethrin


Probit Analysis


Confidence Limits	
	Probability	95% Confidence Limits for Time	95% Confidence Limits for log(Time)a	
		Estimate	Lower Bound	Upper Bound	Estimate	Lower Bound	Upper Bound	
PROBIT	.010	2.368	.486	4.917	.374	-.313	.692	
	.020	3.935	1.090	7.150	.595	.038	.854	
	.030	5.431	1.818	9.081	.735	.260	.958	
	.040	6.920	2.668	10.884	.840	.426	1.037	
	.050	8.428	3.640	12.628	.926	.561	1.101	
	.060	9.968	4.736	14.347	.999	.675	1.157	
	.070	11.548	5.957	16.069	1.063	.775	1.206	
	.080	13.174	7.305	17.811	1.120	.864	1.251	
	.090	14.851	8.778	19.592	1.172	.943	1.292	
	.100	16.583	10.376	21.431	1.220	1.016	1.331	
	.150	26.179	19.925	32.328	1.418	1.299	1.510	
	.200	37.633	30.466	49.233	1.576	1.484	1.692	
	.250	51.378	40.761	75.994	1.711	1.610	1.881	
	.300	67.951	51.405	115.571	1.832	1.711	2.063	
	.350	88.047	63.026	172.353	1.945	1.800	2.236	
	.400	112.585	76.087	253.116	2.051	1.881	2.403	
	.450	142.817	91.046	368.110	2.155	1.959	2.566	
	.500	180.484	108.459	533.053	2.256	2.035	2.727	
	.550	228.087	129.062	772.749	2.358	2.111	2.888	
	.600	289.333	153.887	1127.834	2.461	2.187	3.052	
	.650	369.969	184.464	1668.119	2.568	2.266	3.222	
	.700	479.382	223.176	2521.011	2.681	2.349	3.402	
	.750	634.023	274.000	3938.233	2.802	2.438	3.595	
	.800	865.596	344.194	6474.296	2.937	2.537	3.811	
	.850	1244.284	448.843	11561.284	3.095	2.652	4.063	
	.900	1964.361	626.575	23990.021	3.293	2.797	4.380	
	.910	2193.397	679.111	28617.124	3.341	2.832	4.457	
	.920	2472.576	741.174	34661.354	3.393	2.870	4.540	
	.930	2820.733	815.962	42791.733	3.450	2.912	4.631	
	.940	3267.829	908.420	54147.504	3.514	2.958	4.734	
	.950	3864.867	1026.692	70822.711	3.587	3.011	4.850	
	.960	4707.101	1185.420	97089.414	3.673	3.074	4.987	
	.970	5998.065	1414.501	143092.366	3.778	3.151	5.156	
	.980	8278.142	1788.843	239643.510	3.918	3.253	5.380	
	.990	13755.198	2589.572	540253.396	4.138	3.413	5.733	

a. Logarithm base = 10.	


Alimosho Bendiocarb


Probit Analysis


Confidence Limits	
	Probability	95% Confidence Limits for Time	95% Confidence Limits for log(Time)b	
		Estimate	Lower Bound	Upper Bound	Estimate	Lower Bound	Upper Bound	
PROBITa	.010	5.489	2.511	8.296	.740	.400	.919	
	.020	6.502	3.216	9.458	.813	.507	.976	
	.030	7.239	3.761	10.282	.860	.575	1.012	
	.040	7.849	4.230	10.952	.895	.626	1.040	
	.050	8.382	4.653	11.532	.923	.668	1.062	
	.060	8.864	5.046	12.051	.948	.703	1.081	
	.070	9.310	5.416	12.527	.969	.734	1.098	
	.080	9.727	5.771	12.971	.988	.761	1.113	
	.090	10.124	6.112	13.390	1.005	.786	1.127	
	.100	10.503	6.444	13.788	1.021	.809	1.140	
	.150	12.230	8.009	15.590	1.087	.904	1.193	
	.200	13.803	9.502	17.222	1.140	.978	1.236	
	.250	15.312	10.981	18.794	1.185	1.041	1.274	
	.300	16.808	12.479	20.370	1.226	1.096	1.309	
	.350	18.325	14.015	21.999	1.263	1.147	1.342	
	.400	19.890	15.607	23.728	1.299	1.193	1.375	
	.450	21.531	17.265	25.608	1.333	1.237	1.408	
	.500	23.279	19.002	27.701	1.367	1.279	1.442	
	.550	25.168	20.831	30.086	1.401	1.319	1.478	
	.600	27.245	22.769	32.863	1.435	1.357	1.517	
	.650	29.572	24.844	36.173	1.471	1.395	1.558	
	.700	32.240	27.107	40.215	1.508	1.433	1.604	
	.750	35.389	29.638	45.301	1.549	1.472	1.656	
	.800	39.260	32.582	51.968	1.594	1.513	1.716	
	.850	44.309	36.216	61.272	1.646	1.559	1.787	
	.900	51.594	41.164	75.757	1.713	1.615	1.879	
	.910	53.526	42.432	79.789	1.729	1.628	1.902	
	.920	55.707	43.844	84.431	1.746	1.642	1.927	
	.930	58.208	45.440	89.869	1.765	1.657	1.954	
	.940	61.135	47.280	96.381	1.786	1.675	1.984	
	.950	64.652	49.455	104.416	1.811	1.694	2.019	
	.960	69.044	52.121	114.755	1.839	1.717	2.060	
	.970	74.854	55.573	128.932	1.874	1.745	2.110	
	.980	83.342	60.482	150.615	1.921	1.782	2.178	
	.990	98.715	69.043	192.629	1.994	1.839	2.285	

a. A heterogeneity factor is used.	
b. Logarithm base = 10.	


Badagry DDT


Probit Analysis


Confidence Limits	
	Probability	95% Confidence Limits for Time	95% Confidence Limits for log(Time)a	
		Estimate	Lower Bound	Upper Bound	Estimate	Lower Bound	Upper Bound	
PROBIT	.010	9.266	6.750	11.592	.967	.829	1.064	
	.020	11.416	8.689	13.867	1.058	.939	1.142	
	.030	13.033	10.194	15.544	1.115	1.008	1.192	
	.040	14.398	11.491	16.943	1.158	1.060	1.229	
	.050	15.613	12.664	18.178	1.193	1.103	1.260	
	.060	16.728	13.753	19.305	1.223	1.138	1.286	
	.070	17.771	14.781	20.355	1.250	1.170	1.309	
	.080	18.759	15.764	21.348	1.273	1.198	1.329	
	.090	19.706	16.711	22.297	1.295	1.223	1.348	
	.100	20.620	17.629	23.213	1.314	1.246	1.366	
	.150	24.877	21.940	27.496	1.396	1.341	1.439	
	.200	28.879	25.985	31.605	1.461	1.415	1.500	
	.250	32.821	29.892	35.798	1.516	1.476	1.554	
	.300	36.818	33.725	40.240	1.566	1.528	1.605	
	.350	40.955	37.540	45.056	1.612	1.574	1.654	
	.400	45.309	41.403	50.347	1.656	1.617	1.702	
	.450	49.962	45.389	56.216	1.699	1.657	1.750	
	.500	55.008	49.581	62.792	1.740	1.695	1.798	
	.550	60.564	54.077	70.246	1.782	1.733	1.847	
	.600	66.783	58.994	78.819	1.825	1.771	1.897	
	.650	73.884	64.487	88.861	1.869	1.809	1.949	
	.700	82.186	70.779	100.907	1.915	1.850	2.004	
	.750	92.195	78.210	115.817	1.965	1.893	2.064	
	.800	104.780	87.358	135.102	2.020	1.941	2.131	
	.850	121.635	99.326	161.758	2.085	1.997	2.209	
	.900	146.745	116.671	203.012	2.167	2.067	2.308	
	.910	153.550	121.286	214.478	2.186	2.084	2.331	
	.920	161.300	126.505	227.676	2.208	2.102	2.357	
	.930	170.275	132.498	243.134	2.231	2.122	2.386	
	.940	180.890	139.523	261.651	2.257	2.145	2.418	
	.950	193.806	147.984	284.508	2.287	2.170	2.454	
	.960	210.164	158.576	313.941	2.323	2.200	2.497	
	.970	232.178	172.632	354.352	2.366	2.237	2.549	
	.980	265.052	193.246	416.274	2.423	2.286	2.619	
	.990	326.568	230.808	536.651	2.514	2.363	2.730	

a. Logarithm base = 10.	


Badagry permethrin

Probit Analysis


Confidence Limits	
	Probability	95% Confidence Limits for Time	95% Confidence Limits for log(Time)a	
		Estimate	Lower Bound	Upper Bound	Estimate	Lower Bound	Upper Bound	
PROBIT	.010	9.699	7.507	11.731	.987	.875	1.069	
	.020	11.630	9.293	13.751	1.066	.968	1.138	
	.030	13.050	10.637	15.214	1.116	1.027	1.182	
	.040	14.231	11.773	16.420	1.153	1.071	1.215	
	.050	15.271	12.783	17.475	1.184	1.107	1.242	
	.060	16.215	13.708	18.428	1.210	1.137	1.265	
	.070	17.091	14.573	19.309	1.233	1.164	1.286	
	.080	17.915	15.391	20.137	1.253	1.187	1.304	
	.090	18.699	16.173	20.922	1.272	1.209	1.321	
	.100	19.451	16.927	21.675	1.289	1.229	1.336	
	.150	22.901	20.406	25.129	1.360	1.310	1.400	
	.200	26.073	23.617	28.331	1.416	1.373	1.452	
	.250	29.143	26.701	31.485	1.465	1.427	1.498	
	.300	32.207	29.730	34.712	1.508	1.473	1.540	
	.350	35.333	32.749	38.104	1.548	1.515	1.581	
	.400	38.580	35.800	41.742	1.586	1.554	1.621	
	.450	42.004	38.929	45.701	1.623	1.590	1.660	
	.500	45.671	42.189	50.066	1.660	1.625	1.700	
	.550	49.658	45.645	54.939	1.696	1.659	1.740	
	.600	54.066	49.381	60.459	1.733	1.694	1.781	
	.650	59.033	53.505	66.820	1.771	1.728	1.825	
	.700	64.763	58.172	74.318	1.811	1.765	1.871	
	.750	71.572	63.616	83.420	1.855	1.804	1.921	
	.800	79.999	70.231	94.941	1.903	1.847	1.977	
	.850	91.083	78.760	110.468	1.959	1.896	2.043	
	.900	107.236	90.913	133.758	2.030	1.959	2.126	
	.910	111.549	94.110	140.096	2.047	1.974	2.146	
	.920	116.431	97.708	147.327	2.066	1.990	2.168	
	.930	122.046	101.819	155.716	2.087	2.008	2.192	
	.940	128.638	106.612	165.657	2.109	2.028	2.219	
	.950	136.593	112.348	177.781	2.135	2.051	2.250	
	.960	146.568	119.476	193.174	2.166	2.077	2.286	
	.970	159.836	128.853	213.951	2.204	2.110	2.330	
	.980	179.351	142.451	245.097	2.254	2.154	2.389	
	.990	215.057	166.823	303.704	2.333	2.222	2.482	

a. Logarithm base = 10.	


Badagry Bendiocarb


Probit Analysis


Confidence Limits	
	Probability	95% Confidence Limits for Time	95% Confidence Limits for log(Time)b	
		Estimate	Lower Bound	Upper Bound	Estimate	Lower Bound	Upper Bound	
PROBITa	.010	6.815	4.638	8.735	.833	.666	.941	
	.020	7.755	5.474	9.723	.890	.738	.988	
	.030	8.416	6.079	10.410	.925	.784	1.017	
	.040	8.951	6.576	10.961	.952	.818	1.040	
	.050	9.411	7.010	11.432	.974	.846	1.058	
	.060	9.821	7.401	11.849	.992	.869	1.074	
	.070	10.196	7.760	12.229	1.008	.890	1.087	
	.080	10.543	8.097	12.581	1.023	.908	1.100	
	.090	10.869	8.415	12.910	1.036	.925	1.111	
	.100	11.178	8.718	13.222	1.048	.940	1.121	
	.150	12.554	10.086	14.606	1.099	1.004	1.165	
	.200	13.767	11.311	15.826	1.139	1.054	1.199	
	.250	14.901	12.467	16.973	1.173	1.096	1.230	
	.300	15.999	13.589	18.095	1.204	1.133	1.258	
	.350	17.088	14.702	19.222	1.233	1.167	1.284	
	.400	18.190	15.822	20.384	1.260	1.199	1.309	
	.450	19.323	16.963	21.605	1.286	1.229	1.335	
	.500	20.508	18.137	22.914	1.312	1.259	1.360	
	.550	21.766	19.359	24.343	1.338	1.287	1.386	
	.600	23.122	20.647	25.936	1.364	1.315	1.414	
	.650	24.613	22.023	27.748	1.391	1.343	1.443	
	.700	26.289	23.524	29.857	1.420	1.372	1.475	
	.750	28.226	25.202	32.385	1.451	1.401	1.510	
	.800	30.550	27.149	35.536	1.485	1.434	1.551	
	.850	33.503	29.535	39.696	1.525	1.470	1.599	
	.900	37.626	32.744	45.759	1.575	1.515	1.660	
	.910	38.696	33.557	47.375	1.588	1.526	1.676	
	.920	39.893	34.459	49.202	1.601	1.537	1.692	
	.930	41.251	35.474	51.300	1.615	1.550	1.710	
	.940	42.823	36.636	53.757	1.632	1.564	1.730	
	.950	44.690	38.002	56.714	1.650	1.580	1.754	
	.960	46.986	39.662	60.411	1.672	1.598	1.781	
	.970	49.972	41.791	65.306	1.699	1.621	1.815	
	.980	54.238	44.781	72.461	1.734	1.651	1.860	
	.990	61.711	49.897	85.427	1.790	1.698	1.932	

a. A heterogeneity factor is used.	
b. Logarithm base = 10.	


Ibeju-Lekki DDT


Probit Analysis


Confidence Limits	
	Probability	95% Confidence Limits for Time	95% Confidence Limits for log(Time)a	
		Estimate	Lower Bound	Upper Bound	Estimate	Lower Bound	Upper Bound	
PROBIT	.010	11.144	4.732	16.381	1.047	.675	1.214	
	.020	15.858	8.433	21.355	1.200	.926	1.329	
	.030	19.836	12.091	25.428	1.297	1.082	1.405	
	.040	23.474	15.751	29.188	1.371	1.197	1.465	
	.050	26.920	19.389	32.892	1.430	1.288	1.517	
	.060	30.249	22.950	36.715	1.481	1.361	1.565	
	.070	33.505	26.372	40.790	1.525	1.421	1.611	
	.080	36.716	29.605	45.217	1.565	1.471	1.655	
	.090	39.903	32.630	50.052	1.601	1.514	1.699	
	.100	43.081	35.457	55.317	1.634	1.550	1.743	
	.150	59.165	47.629	87.878	1.772	1.678	1.944	
	.200	76.131	58.434	130.834	1.882	1.767	2.117	
	.250	94.516	69.027	185.708	1.976	1.839	2.269	
	.300	114.780	79.878	255.266	2.060	1.902	2.407	
	.350	137.417	91.281	343.419	2.138	1.960	2.536	
	.400	163.012	103.490	455.563	2.212	2.015	2.659	
	.450	192.305	116.770	599.236	2.284	2.067	2.778	
	.500	226.268	131.435	785.204	2.355	2.119	2.895	
	.550	266.228	147.885	1029.287	2.425	2.170	3.013	
	.600	314.069	166.657	1355.560	2.497	2.222	3.132	
	.650	372.567	188.519	1802.310	2.571	2.275	3.256	
	.700	446.045	214.619	2433.894	2.649	2.332	3.386	
	.750	541.678	246.801	3366.614	2.734	2.392	3.527	
	.800	672.485	288.288	4832.508	2.828	2.460	3.684	
	.850	865.333	345.452	7366.242	2.937	2.538	3.867	
	.900	1188.393	433.634	12522.952	3.075	2.637	4.098	
	.910	1283.031	458.095	14235.916	3.108	2.661	4.153	
	.920	1394.403	486.228	16363.593	3.144	2.687	4.214	
	.930	1528.053	519.152	19072.205	3.184	2.715	4.280	
	.940	1692.518	558.555	22630.984	3.229	2.747	4.355	
	.950	1901.803	607.146	27507.742	3.279	2.783	4.439	
	.960	2180.983	669.643	34596.992	3.339	2.826	4.539	
	.970	2580.972	755.333	45864.695	3.412	2.878	4.661	
	.980	3228.483	886.408	66721.236	3.509	2.948	4.824	
	.990	4594.323	1140.569	120469.969	3.662	3.057	5.081	

a. Logarithm base = 10.	


Ibeju-Lekki Permethrin

Probit Analysis


Confidence Limits	
	Probability	95% Confidence Limits for Time	95% Confidence Limits for log(Time)b	
		Estimate	Lower Bound	Upper Bound	Estimate	Lower Bound	Upper Bound	
PROBITa	.010	6.621	.000	15.278	.821	-3.735	1.184	
	.020	10.894	.007	20.603	1.037	-2.161	1.314	
	.030	14.941	.067	25.380	1.174	-1.171	1.404	
	.040	18.950	.366	30.432	1.278	-.437	1.483	
	.050	22.991	1.393	36.661	1.362	.144	1.564	
	.060	27.103	4.061	45.978	1.433	.609	1.663	
	.070	31.310	9.174	63.434	1.496	.963	1.802	
	.080	35.627	15.852	101.577	1.552	1.200	2.007	
	.090	40.069	21.992	184.761	1.603	1.342	2.267	
	.100	44.645	26.873	354.480	1.650	1.429	2.550	
	.150	69.861	42.428	7643.338	1.844	1.628	3.883	
	.200	99.720	54.190	98768.850	1.999	1.734	4.995	
	.250	135.323	65.437	906378.183	2.131	1.816	5.957	
	.300	178.008	76.925	6685741.110	2.250	1.886	6.825	
	.350	229.496	89.032	42750245.424	2.361	1.950	7.631	
	.400	292.060	102.061	249160650.189	2.465	2.009	8.396	
	.450	368.780	116.318	1373245854.885	2.567	2.066	9.138	
	.500	463.935	132.163	7373718694.641	2.666	2.121	9.868	
	.550	583.643	150.057	39622540314.522	2.766	2.176	10.598	
	.600	736.958	170.623	218884501680.268	2.867	2.232	11.340	
	.650	937.865	194.752	1281220250429.017	2.972	2.289	12.108	
	.700	1209.137	223.787	8251674563895.843	3.082	2.350	12.917	
	.750	1590.540	259.893	61611629389337.125	3.202	2.415	13.790	
	.800	2158.407	306.876	578216593403049.900	3.334	2.487	14.762	
	.850	3080.932	372.313	7865924119384262.000	3.489	2.571	15.896	
	.900	4821.027	474.594	210068369778246656.000	3.683	2.676	17.322	
	.910	5371.641	503.216	464471474535122560.000	3.730	2.702	17.667	
	.920	6041.303	536.256	1099839621748868860.000	3.781	2.729	18.041	
	.930	6874.386	575.078	2837800761824249300.000	3.837	2.760	18.453	
	.940	7941.315	621.747	8179889830490199000.000	3.900	2.794	18.913	
	.950	9361.703	679.587	27360832116977672000.000	3.971	2.832	19.437	
	.960	11358.317	754.417	113039804671305660000.000	4.055	2.878	20.053	
	.970	14405.591	857.740	646663772761813200000.000	4.159	2.933	20.811	
	.980	19757.874	1017.223	6570745099167016000000.000	4.296	3.007	21.818	
	.990	32508.640	1330.642	253952867118670950000000.000	4.512	3.124	23.405	

a. A heterogeneity factor is used.	
b. Logarithm base = 10.	


Ibeju-Lekki Bendiocarb


Probit Analysis


Confidence Limits	
	Probability	95% Confidence Limits for Time	95% Confidence Limits for log(Time)b	
		Estimate	Lower Bound	Upper Bound	Estimate	Lower Bound	Upper Bound	
PROBITa	.010	6.733	4.744	8.511	.828	.676	.930	
	.020	7.670	5.586	9.497	.885	.747	.978	
	.030	8.331	6.194	10.184	.921	.792	1.008	
	.040	8.866	6.694	10.735	.948	.826	1.031	
	.050	9.326	7.129	11.206	.970	.853	1.049	
	.060	9.737	7.521	11.625	.988	.876	1.065	
	.070	10.112	7.882	12.006	1.005	.897	1.079	
	.080	10.459	8.219	12.358	1.020	.915	1.092	
	.090	10.786	8.538	12.689	1.033	.931	1.103	
	.100	11.096	8.842	13.001	1.045	.947	1.114	
	.150	12.475	10.211	14.390	1.096	1.009	1.158	
	.200	13.693	11.437	15.615	1.137	1.058	1.194	
	.250	14.832	12.593	16.766	1.171	1.100	1.224	
	.300	15.936	13.716	17.889	1.202	1.137	1.253	
	.350	17.032	14.830	19.018	1.231	1.171	1.279	
	.400	18.141	15.953	20.178	1.259	1.203	1.305	
	.450	19.283	17.097	21.396	1.285	1.233	1.330	
	.500	20.477	18.278	22.697	1.311	1.262	1.356	
	.550	21.745	19.510	24.116	1.337	1.290	1.382	
	.600	23.114	20.812	25.692	1.364	1.318	1.410	
	.650	24.620	22.207	27.479	1.391	1.346	1.439	
	.700	26.313	23.734	29.554	1.420	1.375	1.471	
	.750	28.271	25.446	32.036	1.451	1.406	1.506	
	.800	30.622	27.440	35.122	1.486	1.438	1.546	
	.850	33.612	29.891	39.187	1.526	1.476	1.593	
	.900	37.791	33.201	45.096	1.577	1.521	1.654	
	.910	38.876	34.042	46.669	1.590	1.532	1.669	
	.920	40.091	34.975	48.446	1.603	1.544	1.685	
	.930	41.469	36.026	50.484	1.618	1.557	1.703	
	.940	43.065	37.231	52.871	1.634	1.571	1.723	
	.950	44.961	38.648	55.741	1.653	1.587	1.746	
	.960	47.295	40.374	59.324	1.675	1.606	1.773	
	.970	50.330	42.590	64.065	1.702	1.629	1.807	
	.980	54.669	45.707	70.984	1.738	1.660	1.851	
	.990	62.280	51.055	83.497	1.794	1.708	1.922	

a. A heterogeneity factor is used.	
b. Logarithm base = 10.	


Kosope Permethrion

Probit Analysis


Confidence Limits	
	Probability	95% Confidence Limits for Time	95% Confidence Limits for log(Time)a	
		Estimate	Lower Bound	Upper Bound	Estimate	Lower Bound	Upper Bound	
PROBIT	.010	20.546	15.771	24.274	1.313	1.198	1.385	
	.020	23.594	18.877	27.204	1.373	1.276	1.435	
	.030	25.759	21.146	29.260	1.411	1.325	1.466	
	.040	27.517	23.022	30.921	1.440	1.362	1.490	
	.050	29.035	24.661	32.353	1.463	1.392	1.510	
	.060	30.393	26.140	33.635	1.483	1.417	1.527	
	.070	31.636	27.501	34.810	1.500	1.439	1.542	
	.080	32.792	28.771	35.909	1.516	1.459	1.555	
	.090	33.880	29.968	36.948	1.530	1.477	1.568	
	.100	34.914	31.105	37.943	1.543	1.493	1.579	
	.150	39.538	36.141	42.528	1.597	1.558	1.629	
	.200	43.647	40.436	46.890	1.640	1.607	1.671	
	.250	47.510	44.232	51.324	1.677	1.646	1.710	
	.300	51.270	47.696	55.953	1.710	1.678	1.748	
	.350	55.019	50.963	60.834	1.741	1.707	1.784	
	.400	58.830	54.141	66.014	1.770	1.734	1.820	
	.450	62.768	57.314	71.558	1.798	1.758	1.855	
	.500	66.901	60.554	77.551	1.825	1.782	1.890	
	.550	71.307	63.928	84.110	1.853	1.806	1.925	
	.600	76.080	67.510	91.396	1.881	1.829	1.961	
	.650	81.350	71.391	99.636	1.910	1.854	1.998	
	.700	87.299	75.695	109.165	1.941	1.879	2.038	
	.750	94.208	80.605	120.512	1.974	1.906	2.081	
	.800	102.546	86.423	134.581	2.011	1.937	2.129	
	.850	113.202	93.708	153.111	2.054	1.972	2.185	
	.900	128.196	103.720	180.151	2.108	2.016	2.256	
	.910	132.106	106.290	187.376	2.121	2.026	2.273	
	.920	136.489	109.152	195.557	2.135	2.038	2.291	
	.930	141.476	112.387	204.969	2.151	2.051	2.312	
	.940	147.262	116.110	216.022	2.168	2.065	2.334	
	.950	154.150	120.505	229.362	2.188	2.081	2.361	
	.960	162.655	125.877	246.098	2.211	2.100	2.391	
	.970	173.757	132.807	268.366	2.240	2.123	2.429	
	.980	189.697	142.604	301.133	2.278	2.154	2.479	
	.990	217.840	159.518	361.125	2.338	2.203	2.558	

a. Logarithm base = 10.	


Kisumu DDT

Confidence Limits	
	Probability	95% Confidence Limits for Time	95% Confidence Limits for log(Time)a	
		Estimate	Lower Bound	Upper Bound	Estimate	Lower Bound	Upper Bound	
PROBIT	.010	6.753	3.951	9.425	.829	.597	.974	
	.020	8.507	5.346	11.385	.930	.728	1.056	
	.030	9.849	6.473	12.841	.993	.811	1.109	
	.040	10.997	7.472	14.065	1.041	.873	1.148	
	.050	12.029	8.394	15.150	1.080	.924	1.180	
	.060	12.982	9.265	16.145	1.113	.967	1.208	
	.070	13.881	10.101	17.075	1.142	1.004	1.232	
	.080	14.738	10.910	17.958	1.168	1.038	1.254	
	.090	15.563	11.699	18.804	1.192	1.068	1.274	
	.100	16.364	12.473	19.624	1.214	1.096	1.293	
	.150	20.140	16.207	23.489	1.304	1.210	1.371	
	.200	23.754	19.846	27.251	1.376	1.298	1.435	
	.250	27.367	23.462	31.154	1.437	1.370	1.494	
	.300	31.078	27.075	35.381	1.492	1.433	1.549	
	.350	34.964	30.700	40.091	1.544	1.487	1.603	
	.400	39.100	34.368	45.428	1.592	1.536	1.657	
	.450	43.567	38.135	51.534	1.639	1.581	1.712	
	.500	48.461	42.076	58.579	1.685	1.624	1.768	
	.550	53.905	46.285	66.786	1.732	1.665	1.825	
	.600	60.063	50.878	76.476	1.779	1.707	1.884	
	.650	67.168	56.008	88.126	1.827	1.748	1.945	
	.700	75.567	61.888	102.470	1.878	1.792	2.011	
	.750	85.813	68.848	120.721	1.934	1.838	2.082	
	.800	98.865	77.444	145.043	1.995	1.889	2.161	
	.850	116.605	88.741	179.818	2.067	1.948	2.255	
	.900	143.517	105.216	235.896	2.157	2.022	2.373	
	.910	150.899	109.620	251.913	2.179	2.040	2.401	
	.920	159.349	114.608	270.561	2.202	2.059	2.432	
	.930	169.187	120.348	292.677	2.228	2.080	2.466	
	.940	180.895	127.093	319.540	2.257	2.104	2.505	
	.950	195.240	135.238	353.223	2.291	2.131	2.548	
	.960	213.554	145.466	397.391	2.330	2.163	2.599	
	.970	238.439	159.090	459.377	2.377	2.202	2.662	
	.980	276.065	179.169	557.078	2.441	2.253	2.746	
	.990	347.782	216.025	755.146	2.541	2.335	2.878	

a. Logarithm base = 10.	


Kisumu Permethrin 
Confidence Limits	
	Probability	95% Confidence Limits for Time	95% Confidence Limits for log(Time)b	
		Estimate	Lower Bound	Upper Bound	Estimate	Lower Bound	Upper Bound	
PROBITa	.010	5.567	2.441	7.616	.746	.388	.882	
	.020	6.095	2.887	8.120	.785	.460	.910	
	.030	6.455	3.211	8.460	.810	.507	.927	
	.040	6.741	3.477	8.726	.829	.541	.941	
	.050	6.982	3.710	8.950	.844	.569	.952	
	.060	7.194	3.920	9.147	.857	.593	.961	
	.070	7.385	4.113	9.323	.868	.614	.970	
	.080	7.561	4.294	9.484	.879	.633	.977	
	.090	7.724	4.465	9.634	.888	.650	.984	
	.100	7.878	4.628	9.775	.896	.665	.990	
	.150	8.546	5.365	10.389	.932	.730	1.017	
	.200	9.118	6.026	10.917	.960	.780	1.038	
	.250	9.638	6.650	11.406	.984	.823	1.057	
	.300	10.131	7.257	11.877	1.006	.861	1.075	
	.350	10.610	7.857	12.349	1.026	.895	1.092	
	.400	11.086	8.459	12.834	1.045	.927	1.108	
	.450	11.567	9.068	13.347	1.063	.957	1.125	
	.500	12.060	9.688	13.903	1.081	.986	1.143	
	.550	12.574	10.322	14.522	1.099	1.014	1.162	
	.600	13.119	10.972	15.231	1.118	1.040	1.183	
	.650	13.707	11.640	16.063	1.137	1.066	1.206	
	.700	14.355	12.332	17.068	1.157	1.091	1.232	
	.750	15.089	13.057	18.318	1.179	1.116	1.263	
	.800	15.951	13.837	19.927	1.203	1.141	1.299	
	.850	17.018	14.718	22.114	1.231	1.168	1.345	
	.900	18.462	15.805	25.371	1.266	1.199	1.404	
	.910	18.829	16.066	26.248	1.275	1.206	1.419	
	.920	19.236	16.351	27.243	1.284	1.214	1.435	
	.930	19.693	16.665	28.389	1.294	1.222	1.453	
	.940	20.217	17.017	29.735	1.306	1.231	1.473	
	.950	20.831	17.422	31.359	1.319	1.241	1.496	
	.960	21.576	17.902	33.395	1.334	1.253	1.524	
	.970	22.530	18.500	36.100	1.353	1.267	1.558	
	.980	23.862	19.314	40.065	1.378	1.286	1.603	
	.990	26.125	20.643	47.275	1.417	1.315	1.675	

a. A heterogeneity factor is used.	
b. Logarithm base = 10.	


Kisumu Bendiocarb 
Confidence Limits	
	Probability	95% Confidence Limits for Time	95% Confidence Limits for log(Time)a	
		Estimate	Lower Bound	Upper Bound	Estimate	Lower Bound	Upper Bound	
PROBIT	.010	6.354	4.861	7.697	.803	.687	.886	
	.020	7.200	5.649	8.575	.857	.752	.933	
	.030	7.794	6.213	9.185	.892	.793	.963	
	.040	8.274	6.673	9.674	.918	.824	.986	
	.050	8.685	7.071	10.091	.939	.850	1.004	
	.060	9.051	7.429	10.462	.957	.871	1.020	
	.070	9.385	7.756	10.798	.972	.890	1.033	
	.080	9.695	8.061	11.110	.987	.906	1.046	
	.090	9.985	8.349	11.401	.999	.922	1.057	
	.100	10.260	8.622	11.677	1.011	.936	1.067	
	.150	11.481	9.845	12.898	1.060	.993	1.111	
	.200	12.554	10.930	13.972	1.099	1.039	1.145	
	.250	13.554	11.946	14.977	1.132	1.077	1.175	
	.300	14.520	12.928	15.953	1.162	1.112	1.203	
	.350	15.477	13.898	16.930	1.190	1.143	1.229	
	.400	16.443	14.871	17.929	1.216	1.172	1.254	
	.450	17.434	15.861	18.972	1.241	1.200	1.278	
	.500	18.469	16.880	20.079	1.266	1.227	1.303	
	.550	19.565	17.943	21.278	1.291	1.254	1.328	
	.600	20.745	19.066	22.599	1.317	1.280	1.354	
	.650	22.039	20.272	24.085	1.343	1.307	1.382	
	.700	23.491	21.593	25.795	1.371	1.334	1.412	
	.750	25.165	23.080	27.821	1.401	1.363	1.444	
	.800	27.170	24.815	30.314	1.434	1.395	1.482	
	.850	29.710	26.955	33.563	1.473	1.431	1.526	
	.900	33.245	29.850	38.229	1.522	1.475	1.582	
	.910	34.161	30.587	39.461	1.534	1.486	1.596	
	.920	35.183	31.404	40.848	1.546	1.497	1.611	
	.930	36.343	32.325	42.433	1.560	1.510	1.628	
	.940	37.684	33.381	44.283	1.576	1.524	1.646	
	.950	39.274	34.624	46.497	1.594	1.539	1.667	
	.960	41.227	36.136	49.248	1.615	1.558	1.692	
	.970	43.763	38.079	52.866	1.641	1.581	1.723	
	.980	47.376	40.812	58.107	1.676	1.611	1.764	
	.990	53.686	45.498	67.478	1.730	1.658	1.829	

a. Logarithm base = 10.	
