# Supplementary material for: First report of AChE1 (G119S) mutation and multiple resistance mechanisms in Anopheles gambiae s.s. in Nigeria
Source: Sci Rep. 2020 May 4;10:7482. doi: 10.1038/s41598-020-64412-7 (PMC7198501; doi:10.1038/s41598-020-64412-7)
Supplement: Supplementary file 3 — Supplementary Information 3. [file 41598_2020_64412_MOESM3_ESM.doc]

PROBIT kad OF Exposed WITH Time
  /LOG 10
  /MODEL PROBIT
  /PRINT FREQ CI
  /CRITERIA P(0.15) ITERATE(20) STEPLIMIT(.1).


Probit Analysis


Parameter Estimates	
	Parameter	Estimate	Std. Error	Z	Sig.	95% Confidence Interval	
						Lower Bound	Upper Bound	
PROBITa	Time	1.774	.267	6.632	.000	1.249	2.298	
	Intercept	-3.717	.415	-8.950	.000	-4.133	-3.302	

a. PROBIT model: PROBIT(p) = Intercept + BX (Covariates X are transformed using the base 10.000 logarithm.)	


Chi-Square Tests	
	Chi-Square	dfb	Sig.	
PROBIT	Pearson Goodness-of-Fit Test	4.181	5	.524a	

a. Since the significance level is greater than .150, no heterogeneity factor is used in the calculation of confidence limits.	
b. Statistics based on individual cases differ from statistics based on aggregated cases.	


Cell Counts and Residuals	
	Number	Time	Number of Subjects	Observed Responses	Expected Responses	Residual		
PROBIT	1	1.000	100	3	2.597	.403		
	2	1.176	100	5	5.141	-.141		
	3	1.301	100	9	7.931	1.069		
	4	1.477	100	14	13.623	.377		
	5	1.602	100	15	19.056	-4.056		
	6	1.699	100	20	24.073	-4.073		
	7	1.778	100	35	28.654	6.346		


Confidence Limits	
	Probability	95% Confidence Limits for Time	95% Confidence Limits for log(Time)a	
		Estimate	Lower Bound	Upper Bound	Estimate	Lower Bound	Upper Bound	
PROBIT	.010	6.085	2.876	9.244	.784	.459	.966	
	.020	8.668	4.725	12.217	.938	.674	1.087	
	.030	10.851	6.465	14.606	1.035	.811	1.165	
	.040	12.847	8.174	16.728	1.109	.912	1.223	
	.050	14.739	9.879	18.703	1.168	.995	1.272	
	.060	16.568	11.593	20.593	1.219	1.064	1.314	
	.070	18.357	13.321	22.437	1.264	1.125	1.351	
	.080	20.122	15.065	24.261	1.304	1.178	1.385	
	.090	21.874	16.823	26.089	1.340	1.226	1.416	
	.100	23.622	18.591	27.938	1.373	1.269	1.446	
	.150	32.473	27.396	38.077	1.512	1.438	1.581	
	.200	41.817	35.751	50.789	1.621	1.553	1.706	
	.250	51.950	43.747	66.773	1.716	1.641	1.825	
	.300	63.126	51.793	86.441	1.800	1.714	1.937	
	.350	75.618	60.208	110.453	1.879	1.780	2.043	
	.400	89.750	69.237	139.815	1.953	1.840	2.146	
	.450	105.931	79.113	175.957	2.025	1.898	2.245	
	.500	124.702	90.099	220.901	2.096	1.955	2.344	
	.550	146.799	102.525	277.556	2.167	2.011	2.443	
	.600	173.267	116.834	350.246	2.239	2.068	2.544	
	.650	205.648	133.659	445.659	2.313	2.126	2.649	
	.700	246.342	153.957	574.703	2.392	2.187	2.759	
	.750	299.338	179.267	756.455	2.476	2.254	2.879	
	.800	371.872	212.307	1027.588	2.570	2.327	3.012	
	.850	478.885	258.493	1469.046	2.680	2.412	3.167	
	.900	658.314	331.009	2304.093	2.818	2.520	3.363	
	.910	710.907	351.363	2568.814	2.852	2.546	3.410	
	.920	772.815	374.888	2891.032	2.888	2.574	3.461	
	.930	847.127	402.570	3292.239	2.928	2.605	3.517	
	.940	938.599	435.898	3806.579	2.972	2.639	3.581	
	.950	1055.040	477.276	4492.089	3.023	2.679	3.652	
	.960	1210.427	530.917	5456.983	3.083	2.725	3.737	
	.970	1433.163	605.165	6932.018	3.156	2.782	3.841	
	.980	1793.951	720.134	9528.298	3.254	2.857	3.979	
	.990	2555.678	947.144	15733.384	3.408	2.976	4.197	

a. Logarithm base = 10.	


PROBIT aad OF Exposed WITH Time
  /LOG 10
  /MODEL PROBIT
  /PRINT FREQ CI
  /CRITERIA P(0.15) ITERATE(20) STEPLIMIT(.1).


Probit Analysis


Parameter Estimates	
	Parameter	Estimate	Std. Error	Z	Sig.	95% Confidence Interval	
						Lower Bound	Upper Bound	
PROBITa	Time	2.741	.245	11.203	.000	2.261	3.220	
	Intercept	-4.583	.377	-12.168	.000	-4.960	-4.206	

a. PROBIT model: PROBIT(p) = Intercept + BX (Covariates X are transformed using the base 10.000 logarithm.)	


Chi-Square Tests	
	Chi-Square	dfb	Sig.	
PROBIT	Pearson Goodness-of-Fit Test	6.705	5	.244a	

a. Since the significance level is greater than .150, no heterogeneity factor is used in the calculation of confidence limits.	
b. Statistics based on individual cases differ from statistics based on aggregated cases.	


Cell Counts and Residuals	
	Number	Time	Number of Subjects	Observed Responses	Expected Responses	Residual		
PRBIT	1	1.000	100	1	3.271	-2.271		
	2	1.176	100	8	8.696	-.696		
	3	1.301	100	23	15.451	7.549		
	4	1.477	100	28	29.644	-1.644		
	5	1.602	100	39	42.377	-3.377		
	6	1.699	100	54	52.924	1.076		
	7	1.778	100	61	61.423	-.423		


Confidence Limits	
	Probability	95% Confidence Limits for Time	95% Confidence Limits for log(Time)a	
		Estimate	Lower Bound	Upper Bound	Estimate	Lower Bound	Upper Bound	
PROBIT	.010	6.659	4.666	8.584	.823	.669	.934	
	.020	8.373	6.145	10.454	.923	.789	1.019	
	.030	9.682	7.316	11.851	.986	.864	1.074	
	.040	10.800	8.339	13.027	1.033	.921	1.115	
	.050	11.805	9.274	14.072	1.072	.967	1.148	
	.060	12.733	10.150	15.030	1.105	1.006	1.177	
	.070	13.606	10.984	15.926	1.134	1.041	1.202	
	.080	14.439	11.787	16.777	1.160	1.071	1.225	
	.090	15.241	12.566	17.592	1.183	1.099	1.245	
	.100	16.018	13.327	18.380	1.205	1.125	1.264	
	.150	19.681	16.965	22.082	1.294	1.230	1.344	
	.200	23.181	20.481	25.639	1.365	1.311	1.409	
	.250	26.675	23.976	29.261	1.426	1.380	1.466	
	.300	30.260	27.495	33.097	1.481	1.439	1.520	
	.350	34.011	31.069	37.275	1.532	1.492	1.571	
	.400	37.999	34.733	41.913	1.580	1.541	1.622	
	.450	42.301	38.542	47.127	1.626	1.586	1.673	
	.500	47.012	42.572	53.049	1.672	1.629	1.725	
	.550	52.246	46.917	59.851	1.718	1.671	1.777	
	.600	58.162	51.697	67.772	1.765	1.713	1.831	
	.650	64.982	57.075	77.164	1.813	1.756	1.887	
	.700	73.037	63.282	88.568	1.864	1.801	1.947	
	.750	82.852	70.678	102.862	1.918	1.849	2.012	
	.800	95.342	79.875	121.602	1.979	1.902	2.085	
	.850	112.296	92.050	147.903	2.050	1.964	2.170	
	.900	137.975	109.955	189.367	2.140	2.041	2.277	
	.910	145.011	114.767	201.034	2.161	2.060	2.303	
	.920	153.062	120.230	214.531	2.185	2.080	2.331	
	.930	162.432	126.532	230.429	2.211	2.102	2.363	
	.940	173.576	133.957	249.592	2.239	2.127	2.397	
	.950	187.222	142.951	273.412	2.272	2.155	2.437	
	.960	204.631	154.283	304.336	2.311	2.188	2.483	
	.970	228.267	169.441	347.213	2.358	2.229	2.541	
	.980	263.967	191.900	413.747	2.422	2.283	2.617	
	.990	331.903	233.448	545.540	2.521	2.368	2.737	

a. Logarithm base = 10.	


PROBIT bad OF Exposed WITH Time
  /LOG 10
  /MODEL PROBIT
  /PRINT FREQ CI
  /CRITERIA P(0.15) ITERATE(20) STEPLIMIT(.1).


Probit Analysis


Parameter Estimates	
	Parameter	Estimate	Std. Error	Z	Sig.	95% Confidence Interval	
						Lower Bound	Upper Bound	
PROBITa	Time	3.615	.303	11.941	.000	3.022	4.209	
	Intercept	-6.081	.479	-12.705	.000	-6.559	-5.602	

a. PROBIT model: PROBIT(p) = Intercept + BX (Covariates X are transformed using the base 10.000 logarithm.)	


Chi-Square Tests	
	Chi-Square	dfb	Sig.	
PROBIT	Pearson Goodness-of-Fit Test	3.337	5	.648a	

a. Since the significance level is greater than .150, no heterogeneity factor is used in the calculation of confidence limits.	
b. Statistics based on individual cases differ from statistics based on aggregated cases.	


Cell Counts and Residuals	
	Number	Time	Number of Subjects	Observed Responses	Expected Responses	Residual		
PROBIT	1	1.000	100	0	.685	-.685		
	2	1.176	100	6	3.374	2.626		
	3	1.301	100	7	8.429	-1.429		
	4	1.477	100	22	22.961	-.961		
	5	1.602	100	37	38.652	-1.652		
	6	1.699	100	54	52.471	1.529		
	7	1.778	100	64	63.617	.383		


Confidence Limits	
	Probability	95% Confidence Limits for Time	95% Confidence Limits for log(Time)a	
		Estimate	Lower Bound	Upper Bound	Estimate	Lower Bound	Upper Bound	
PROBIT	.010	10.924	8.484	13.146	1.038	.929	1.119	
	.020	12.995	10.422	15.290	1.114	1.018	1.184	
	.030	14.508	11.871	16.834	1.162	1.075	1.226	
	.040	15.762	13.090	18.102	1.198	1.117	1.258	
	.050	16.861	14.170	19.207	1.227	1.151	1.283	
	.060	17.856	15.157	20.203	1.252	1.181	1.305	
	.070	18.778	16.076	21.123	1.274	1.206	1.325	
	.080	19.643	16.945	21.984	1.293	1.229	1.342	
	.090	20.464	17.773	22.801	1.311	1.250	1.358	
	.100	21.250	18.569	23.582	1.327	1.269	1.373	
	.150	24.841	22.225	27.156	1.395	1.347	1.434	
	.200	28.122	25.571	30.459	1.449	1.408	1.484	
	.250	31.280	28.758	33.707	1.495	1.459	1.528	
	.300	34.418	31.862	37.028	1.537	1.503	1.569	
	.350	37.606	34.933	40.516	1.575	1.543	1.608	
	.400	40.903	38.016	44.251	1.612	1.580	1.646	
	.450	44.368	41.159	48.308	1.647	1.614	1.684	
	.500	48.065	44.418	52.767	1.682	1.648	1.722	
	.550	52.070	47.860	57.727	1.717	1.680	1.761	
	.600	56.481	51.567	63.325	1.752	1.712	1.802	
	.650	61.434	55.644	69.751	1.788	1.745	1.844	
	.700	67.123	60.240	77.295	1.827	1.780	1.888	
	.750	73.856	65.580	86.415	1.868	1.817	1.937	
	.800	82.151	72.040	97.905	1.915	1.858	1.991	
	.850	93.003	80.326	113.310	1.968	1.905	2.054	
	.900	108.716	92.060	136.272	2.036	1.964	2.134	
	.910	112.894	95.134	142.495	2.053	1.978	2.154	
	.920	117.614	98.587	149.582	2.070	1.994	2.175	
	.930	123.033	102.525	157.788	2.090	2.011	2.198	
	.940	129.380	107.105	167.492	2.112	2.030	2.224	
	.950	137.019	112.575	179.297	2.137	2.051	2.254	
	.960	146.573	119.352	194.244	2.166	2.077	2.288	
	.970	159.235	128.238	214.353	2.202	2.108	2.331	
	.980	177.778	141.068	244.366	2.250	2.149	2.388	
	.990	211.483	163.917	300.481	2.325	2.215	2.478	

a. Logarithm base = 10.	


PROBIT iad OF Exposed WITH Time
  /LOG 10
  /MODEL PROBIT
  /PRINT FREQ CI
  /CRITERIA P(0.15) ITERATE(20) STEPLIMIT(.1).


Probit Analysis


Parameter Estimates	
	Parameter	Estimate	Std. Error	Z	Sig.	95% Confidence Interval	
						Lower Bound	Upper Bound	
PROBITa	Time	2.924	.234	12.517	.000	2.466	3.382	
	Intercept	-4.634	.355	-13.063	.000	-4.989	-4.279	

a. PROBIT model: PROBIT(p) = Intercept + BX (Covariates X are transformed using the base 10.000 logarithm.)	


Chi-Square Tests	
	Chi-Square	dfb	Sig.	
PROBIT	Pearson Goodness-of-Fit Test	5.124	5	.401a	

a. Since the significance level is greater than .150, no heterogeneity factor is used in the calculation of confidence limits.	
b. Statistics based on individual cases differ from statistics based on aggregated cases.	


Confidence Limits	
	Probability	95% Confidence Limits for Time	95% Confidence Limits for log(Time)a	
		Estimate	Lower Bound	Upper Bound	Estimate	Lower Bound	Upper Bound	
PROBIT	.010	6.154	4.481	7.784	.789	.651	.891	
	.020	7.628	5.769	9.389	.882	.761	.973	
	.030	8.741	6.770	10.577	.942	.831	1.024	
	.040	9.684	7.635	11.572	.986	.883	1.063	
	.050	10.526	8.418	12.452	1.022	.925	1.095	
	.060	11.300	9.146	13.254	1.053	.961	1.122	
	.070	12.025	9.835	14.002	1.080	.993	1.146	
	.080	12.713	10.495	14.710	1.104	1.021	1.168	
	.090	13.374	11.132	15.385	1.126	1.047	1.187	
	.100	14.012	11.751	16.036	1.147	1.070	1.205	
	.150	16.996	14.686	19.061	1.230	1.167	1.280	
	.200	19.814	17.496	21.914	1.297	1.243	1.341	
	.250	22.601	20.284	24.758	1.354	1.307	1.394	
	.300	25.436	23.102	27.698	1.405	1.364	1.442	
	.350	28.381	25.985	30.825	1.453	1.415	1.489	
	.400	31.489	28.960	34.227	1.498	1.462	1.534	
	.450	34.820	32.062	37.994	1.542	1.506	1.580	
	.500	38.442	35.336	42.227	1.585	1.548	1.626	
	.550	42.440	38.849	47.049	1.628	1.589	1.673	
	.600	46.930	42.688	52.621	1.671	1.630	1.721	
	.650	52.070	46.976	59.173	1.717	1.672	1.772	
	.700	58.097	51.891	67.055	1.764	1.715	1.826	
	.750	65.386	57.708	76.830	1.815	1.761	1.886	
	.800	74.583	64.890	89.492	1.873	1.812	1.952	
	.850	86.950	74.327	107.009	1.939	1.871	2.029	
	.900	105.463	88.087	134.130	2.023	1.945	2.128	
	.910	110.496	91.766	141.669	2.043	1.963	2.151	
	.920	116.237	95.931	150.346	2.065	1.982	2.177	
	.930	122.894	100.726	160.510	2.090	2.003	2.206	
	.940	130.780	106.359	172.684	2.117	2.027	2.237	
	.950	140.395	113.162	187.711	2.147	2.054	2.273	
	.960	152.597	121.704	207.060	2.184	2.085	2.316	
	.970	169.061	133.082	233.621	2.228	2.124	2.369	
	.980	193.729	149.851	274.314	2.287	2.176	2.438	
	.990	240.118	180.630	353.398	2.380	2.257	2.548	

a. Logarithm base = 10.	


PROBIT aap OF Exposed WITH Time
  /LOG 10
  /MODEL PROBIT
  /PRINT FREQ CI
  /CRITERIA P(0.15) ITERATE(20) STEPLIMIT(.1).


Probit Analysis


Convergence Information	
	Number of Iterations	Optimal Solution Found	
PROBIT	11	Yes	


Parameter Estimates	
	Parameter	Estimate	Std. Error	Z	Sig.	95% Confidence Interval	
						Lower Bound	Upper Bound	
PROBITa	Time	2.914	.230	12.647	.000	2.463	3.366	
	Intercept	-4.578	.349	-13.116	.000	-4.927	-4.228	

a. PROBIT model: PROBIT(p) = Intercept + BX (Covariates X are transformed using the base 10.000 logarithm.)	


Chi-Square Tests	
	Chi-Square	dfb	Sig.	
PROBIT	Pearson Goodness-of-Fit Test	1.493	5	.914a	

a. Since the significance level is greater than .150, no heterogeneity factor is used in the calculation of confidence limits.	
b. Statistics based on individual cases differ from statistics based on aggregated cases.	

Confidence Limits	
	Probability	95% Confidence Limits for Time	95% Confidence Limits for log(Time)a	
		Estimate	Lower Bound	Upper Bound	Estimate	Lower Bound	Upper Bound	
PROBIT	.010	5.922	4.305	7.503	.772	.634	.875	
	.020	7.345	5.545	9.057	.866	.744	.957	
	.030	8.420	6.510	10.209	.925	.814	1.009	
	.040	9.332	7.342	11.173	.970	.866	1.048	
	.050	10.146	8.097	12.026	1.006	.908	1.080	
	.060	10.894	8.798	12.805	1.037	.944	1.107	
	.070	11.596	9.462	13.531	1.064	.976	1.131	
	.080	12.262	10.098	14.217	1.089	1.004	1.153	
	.090	12.901	10.713	14.872	1.111	1.030	1.172	
	.100	13.519	11.310	15.503	1.131	1.053	1.190	
	.150	16.408	14.143	18.438	1.215	1.151	1.266	
	.200	19.139	16.859	21.203	1.282	1.227	1.326	
	.250	21.840	19.559	23.956	1.339	1.291	1.379	
	.300	24.590	22.294	26.798	1.391	1.348	1.428	
	.350	27.446	25.098	29.816	1.438	1.400	1.474	
	.400	30.463	28.000	33.093	1.484	1.447	1.520	
	.450	33.696	31.030	36.719	1.528	1.492	1.565	
	.500	37.213	34.232	40.794	1.571	1.534	1.611	
	.550	41.098	37.669	45.437	1.614	1.576	1.657	
	.600	45.460	41.425	50.805	1.658	1.617	1.706	
	.650	50.456	45.621	57.122	1.703	1.659	1.757	
	.700	56.317	50.430	64.725	1.751	1.703	1.811	
	.750	63.407	56.122	74.160	1.802	1.749	1.870	
	.800	72.358	63.151	86.385	1.859	1.800	1.936	
	.850	84.398	72.390	103.305	1.926	1.860	2.014	
	.900	102.433	85.870	129.514	2.010	1.934	2.112	
	.910	107.339	89.475	136.800	2.031	1.952	2.136	
	.920	112.934	93.558	145.189	2.053	1.971	2.162	
	.930	119.424	98.258	155.014	2.077	1.992	2.190	
	.940	127.114	103.782	166.785	2.104	2.016	2.222	
	.950	136.491	110.454	181.316	2.135	2.043	2.258	
	.960	148.394	118.835	200.028	2.171	2.075	2.301	
	.970	164.460	130.002	225.720	2.216	2.114	2.354	
	.980	188.542	146.468	265.088	2.275	2.166	2.423	
	.990	233.855	176.713	341.621	2.369	2.247	2.534	

a. Logarithm base = 10.	


PROBIT bap OF Exposed WITH Time
  /LOG 10
  /MODEL PROBIT
  /PRINT FREQ CI
  /CRITERIA P(0.15) ITERATE(20) STEPLIMIT(.1).


Probit Analysis


Parameter Estimates	
	Parameter	Estimate	Std. Error	Z	Sig.	95% Confidence Interval	
						Lower Bound	Upper Bound	
PROBITa	Time	4.751	.326	14.575	.000	4.112	5.390	
	Intercept	-7.490	.509	-14.702	.000	-8.000	-6.981	

a. PROBIT model: PROBIT(p) = Intercept + BX (Covariates X are transformed using the base 10.000 logarithm.)	


Chi-Square Tests	
	Chi-Square	dfb	Sig.	
PROBIT	Pearson Goodness-of-Fit Test	16.781	5	.005a	

a. Since the significance level is less than .150, a heterogeneity factor is used in the calculation of confidence limits.	
b. Statistics based on individual cases differ from statistics based on aggregated cases.	


Confidence Limits	
	Probability	95% Confidence Limits for Time	95% Confidence Limits for log(Time)b	
		Estimate	Lower Bound	Upper Bound	Estimate	Lower Bound	Upper Bound	
PROBITa	.010	12.213	7.135	16.289	1.087	.853	1.212	
	.020	13.938	8.642	18.062	1.144	.937	1.257	
	.030	15.157	9.755	19.295	1.181	.989	1.285	
	.040	16.143	10.683	20.284	1.208	1.029	1.307	
	.050	16.993	11.499	21.130	1.230	1.061	1.325	
	.060	17.751	12.240	21.883	1.249	1.088	1.340	
	.070	18.443	12.927	22.570	1.266	1.112	1.354	
	.080	19.086	13.573	23.206	1.281	1.133	1.366	
	.090	19.691	14.186	23.804	1.294	1.152	1.377	
	.100	20.264	14.772	24.371	1.307	1.169	1.387	
	.150	22.820	17.440	26.914	1.358	1.242	1.430	
	.200	25.079	19.847	29.200	1.399	1.298	1.465	
	.250	27.195	22.115	31.401	1.434	1.345	1.497	
	.300	29.246	24.302	33.615	1.466	1.386	1.527	
	.350	31.285	26.441	35.914	1.495	1.422	1.555	
	.400	33.352	28.553	38.364	1.523	1.456	1.584	
	.450	35.481	30.654	41.030	1.550	1.486	1.613	
	.500	37.708	32.764	43.980	1.576	1.515	1.643	
	.550	40.076	34.907	47.293	1.603	1.543	1.675	
	.600	42.634	37.117	51.067	1.630	1.570	1.708	
	.650	45.450	39.441	55.437	1.658	1.596	1.744	
	.700	48.619	41.943	60.597	1.687	1.623	1.782	
	.750	52.287	44.721	66.856	1.718	1.651	1.825	
	.800	56.698	47.930	74.747	1.754	1.681	1.874	
	.850	62.311	51.854	85.305	1.795	1.715	1.931	
	.900	70.171	57.124	100.959	1.846	1.757	2.004	
	.910	72.213	58.458	105.182	1.859	1.767	2.022	
	.920	74.499	59.938	109.980	1.872	1.778	2.041	
	.930	77.097	61.600	115.523	1.887	1.790	2.063	
	.940	80.105	63.504	122.059	1.904	1.803	2.087	
	.950	83.679	65.738	129.984	1.923	1.818	2.114	
	.960	88.083	68.452	139.979	1.945	1.835	2.146	
	.970	93.815	71.927	153.359	1.972	1.857	2.186	
	.980	102.017	76.797	173.199	2.009	1.885	2.239	
	.990	116.425	85.102	209.927	2.066	1.930	2.322	

a. A heterogeneity factor is used.	
b. Logarithm base = 10.	


PROBIT iap OF Exposed WITH Time
  /LOG 10
  /MODEL PROBIT
  /PRINT FREQ CI
  /CRITERIA P(0.15) ITERATE(20) STEPLIMIT(.1).


Probit Analysis


Parameter Estimates	
	Parameter	Estimate	Std. Error	Z	Sig.	95% Confidence Interval	
						Lower Bound	Upper Bound	
PROBITa	Time	3.323	.228	14.587	.000	2.876	3.769	
	Intercept	-4.671	.332	-14.081	.000	-5.003	-4.340	

a. PROBIT model: PROBIT(p) = Intercept + BX (Covariates X are transformed using the base 10.000 logarithm.)	


Chi-Square Tests	
	Chi-Square	dfb	Sig.	
PROBIT	Pearson Goodness-of-Fit Test	4.631	5	.463a	

a. Since the significance level is greater than .150, no heterogeneity factor is used in the calculation of confidence limits.	
b. Statistics based on individual cases differ from statistics based on aggregated cases.	


Confidence Limits	
	Probability	95% Confidence Limits for Time	95% Confidence Limits for log(Time)a	
		Estimate	Lower Bound	Upper Bound	Estimate	Lower Bound	Upper Bound	
PROBIT	.010	5.079	3.866	6.273	.706	.587	.797	
	.020	6.136	4.802	7.420	.788	.681	.870	
	.030	6.917	5.509	8.256	.840	.741	.917	
	.040	7.570	6.109	8.947	.879	.786	.952	
	.050	8.146	6.643	9.552	.911	.822	.980	
	.060	8.670	7.135	10.101	.938	.853	1.004	
	.070	9.158	7.595	10.608	.962	.881	1.026	
	.080	9.618	8.031	11.084	.983	.905	1.045	
	.090	10.057	8.450	11.537	1.002	.927	1.062	
	.100	10.478	8.854	11.970	1.020	.947	1.078	
	.150	12.418	10.736	13.952	1.094	1.031	1.145	
	.200	14.213	12.501	15.774	1.153	1.097	1.198	
	.250	15.958	14.232	17.542	1.203	1.153	1.244	
	.300	17.707	15.973	19.318	1.248	1.203	1.286	
	.350	19.499	17.756	21.148	1.290	1.249	1.325	
	.400	21.367	19.606	23.075	1.330	1.292	1.363	
	.450	23.343	21.545	25.144	1.368	1.333	1.400	
	.500	25.468	23.602	27.409	1.406	1.373	1.438	
	.550	27.785	25.806	29.932	1.444	1.412	1.476	
	.600	30.356	28.203	32.798	1.482	1.450	1.516	
	.650	33.263	30.853	36.120	1.522	1.489	1.558	
	.700	36.629	33.852	40.061	1.564	1.530	1.603	
	.750	40.644	37.349	44.878	1.609	1.572	1.652	
	.800	45.635	41.600	51.011	1.659	1.619	1.708	
	.850	52.231	47.093	59.324	1.718	1.673	1.773	
	.900	61.902	54.953	71.856	1.792	1.740	1.856	
	.910	64.495	57.028	75.276	1.810	1.756	1.877	
	.920	67.434	59.367	79.182	1.829	1.774	1.899	
	.930	70.822	62.045	83.717	1.850	1.793	1.923	
	.940	74.806	65.174	89.099	1.874	1.814	1.950	
	.950	79.625	68.928	95.669	1.901	1.838	1.981	
	.960	85.685	73.607	104.021	1.933	1.867	2.017	
	.970	93.770	79.786	115.312	1.972	1.902	2.062	
	.980	105.711	88.791	132.268	2.024	1.948	2.121	
	.990	127.693	105.054	164.256	2.106	2.021	2.216	

a. Logarithm base = 10.	


GET
  FILE='C:\Users\HP\Documents\thesis materials\Data and analyses\PBO data.sav'.
DATASET NAME DataSet1 WINDOW=FRONT.
PROBIT kap OF Exposed WITH Time
  /LOG 10
  /MODEL PROBIT
  /PRINT FREQ CI
  /CRITERIA P(.15) ITERATE(20) STEPLIMIT(.1).


Probit Analysis


[DataSet1] C:\Users\HP\Documents\thesis materials\Data and analyses\PBO data.sav


Parameter Estimates	
	Parameter	Estimate	Std. Error	Z	Sig.	95% Confidence Interval	
						Lower Bound	Upper Bound	
PROBITa	Time	2.200	.243	9.059	.000	1.724	2.676	
	Intercept	-4.033	.375	-10.759	.000	-4.408	-3.658	

a. PROBIT model: PROBIT(p) = Intercept + BX (Covariates X are transformed using the base 10.000 logarithm.)	


Chi-Square Tests	
	Chi-Square	dfb	Sig.	
PROBIT	Pearson Goodness-of-Fit Test	8.291	5	.141a	

a. Since the significance level is less than .150, a heterogeneity factor is used in the calculation of confidence limits.	
b. Statistics based on individual cases differ from statistics based on aggregated cases.	


Confidence Limits	
	Probability	95% Confidence Limits for Time	95% Confidence Limits for log(Time)b	
		Estimate	Lower Bound	Upper Bound	Estimate	Lower Bound	Upper Bound	
PROBITa	.010	5.970	2.144	9.763	.776	.331	.990	
	.020	7.942	3.342	12.099	.900	.524	1.083	
	.030	9.518	4.425	13.880	.979	.646	1.142	
	.040	10.907	5.460	15.404	1.038	.737	1.188	
	.050	12.185	6.473	16.778	1.086	.811	1.225	
	.060	13.390	7.478	18.057	1.127	.874	1.257	
	.070	14.544	8.480	19.271	1.163	.928	1.285	
	.080	15.662	9.485	20.440	1.195	.977	1.310	
	.090	16.752	10.494	21.579	1.224	1.021	1.334	
	.100	17.823	11.511	22.699	1.251	1.061	1.356	
	.150	23.037	16.711	28.271	1.362	1.223	1.451	
	.200	28.249	22.036	34.328	1.451	1.343	1.536	
	.250	33.649	27.303	41.490	1.527	1.436	1.618	
	.300	39.374	32.392	50.260	1.595	1.510	1.701	
	.350	45.545	37.344	61.008	1.658	1.572	1.785	
	.400	52.292	42.297	74.094	1.718	1.626	1.870	
	.450	59.771	47.403	90.007	1.776	1.676	1.954	
	.500	68.174	52.808	109.458	1.834	1.723	2.039	
	.550	77.758	58.663	133.489	1.891	1.768	2.125	
	.600	88.878	65.148	163.645	1.949	1.814	2.214	
	.650	102.045	72.495	202.295	2.009	1.860	2.306	
	.700	118.038	81.039	253.248	2.072	1.909	2.404	
	.750	138.120	91.300	323.047	2.140	1.960	2.509	
	.800	164.527	104.170	424.012	2.216	2.018	2.627	
	.850	201.746	121.373	582.678	2.305	2.084	2.765	
	.900	260.760	146.972	870.034	2.416	2.167	2.940	
	.910	277.432	153.906	958.608	2.443	2.187	2.982	
	.920	296.753	161.804	1065.126	2.472	2.209	3.027	
	.930	319.556	170.948	1196.019	2.505	2.233	3.078	
	.940	347.099	181.762	1361.382	2.540	2.260	3.134	
	.950	381.423	194.921	1578.164	2.581	2.290	3.198	
	.960	426.109	211.587	1877.497	2.630	2.325	3.274	
	.970	488.282	234.018	2324.615	2.689	2.369	3.366	
	.980	585.194	267.520	3088.432	2.767	2.427	3.490	
	.990	778.449	330.233	4834.269	2.891	2.519	3.684	

a. A heterogeneity factor is used.	
b. Logarithm base = 10.	
